# Supplementary material for: Transcription and chromatin-based surveillance mechanism controls suppression of cryptic antisense transcription
Source: Cell Rep. 2021 Sep 7;36(10):109671. doi: 10.1016/j.celrep.2021.109671 (PMC8441049; doi:10.1016/j.celrep.2021.109671)
Supplement: Document S1. Figures S1–S4 [file mmc1.pdf]

Cell Reports, Volume *36*

Supplemental information

Transcription and chromatin-based surveillance mechanism controls suppression of cryptic antisense transcription

Dong-Hyuk Heo, Krzysztof Kuś, Pawel Grzechnik, Sue Mei Tan-Wong, Adrien Birot, Tea Kecman, Soren Nielsen, Nikolay Zenkin, and Lidia Vasiljeva

Figure S1

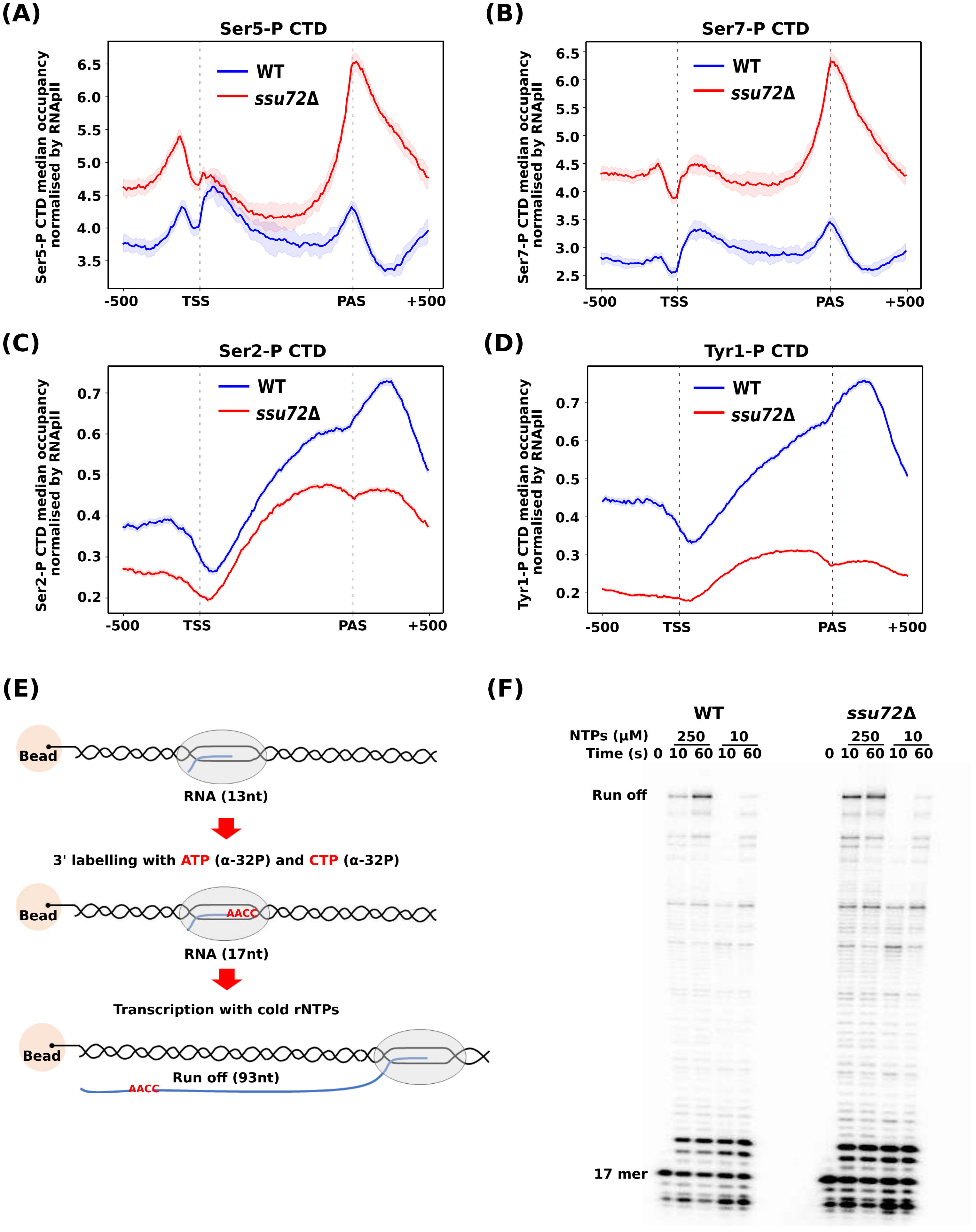
(A)
Ser5-P CTD
Ser5-P CTD median occupancy normalised by RNApII
WT
ssu72Δ
-500
TSS
PAS
+500
(B)
Ser7-P CTD
Ser7-P CTD median occupancy normalised by RNApII
(C)
Ser2-P CTD
Ser2-P CTD median occupancy normalised by RNApII
(D)
Tyr1-P CTD
Tyr1-P CTD median occupancy normalised by RNApII
(E)
Bead
RNA (13nt)
3' labelling with ATP (α-32P) and CTP (α-32P)
AACC
RNA (17nt)
Transcription with cold rNTPs
Run off (93nt)
(F)
WT
ssu72Δ
NTPs (μM) 250 10
Time (s) 0 10 60 10 60
Run off
17 mer

Figure S1. Ssu72 modifies RNApII CTD phosphorylation pattern *in vivo* but changes do not alter enzymatic properties of purified RNApII *in vitro*. Related to Figures 1 and 2.

(A to D) Metagene profiles of RNApII CTD phosphorylation (Ser5-P in (A), Ser7-P in (B), Ser2-P in (C), and Tyr1-P in (D)) in WT (blue) and *ssu72*Δ (red) strains generated using 10-bp bins within ±500 bp around TSS and PAS. The signal was normalised by RNApII occupancy (8WG16 antibody). The shaded regions represent 95% confidence intervals.

(E) A schematic diagram describing *in vitro* transcription assay.

(F) RNAs transcribed by RNApII purified from WT and *ssu72*Δ strains were resolved in 10% denaturating UREA-TBE acrylamide gel and detected by phosphorimaging.

Figure S2

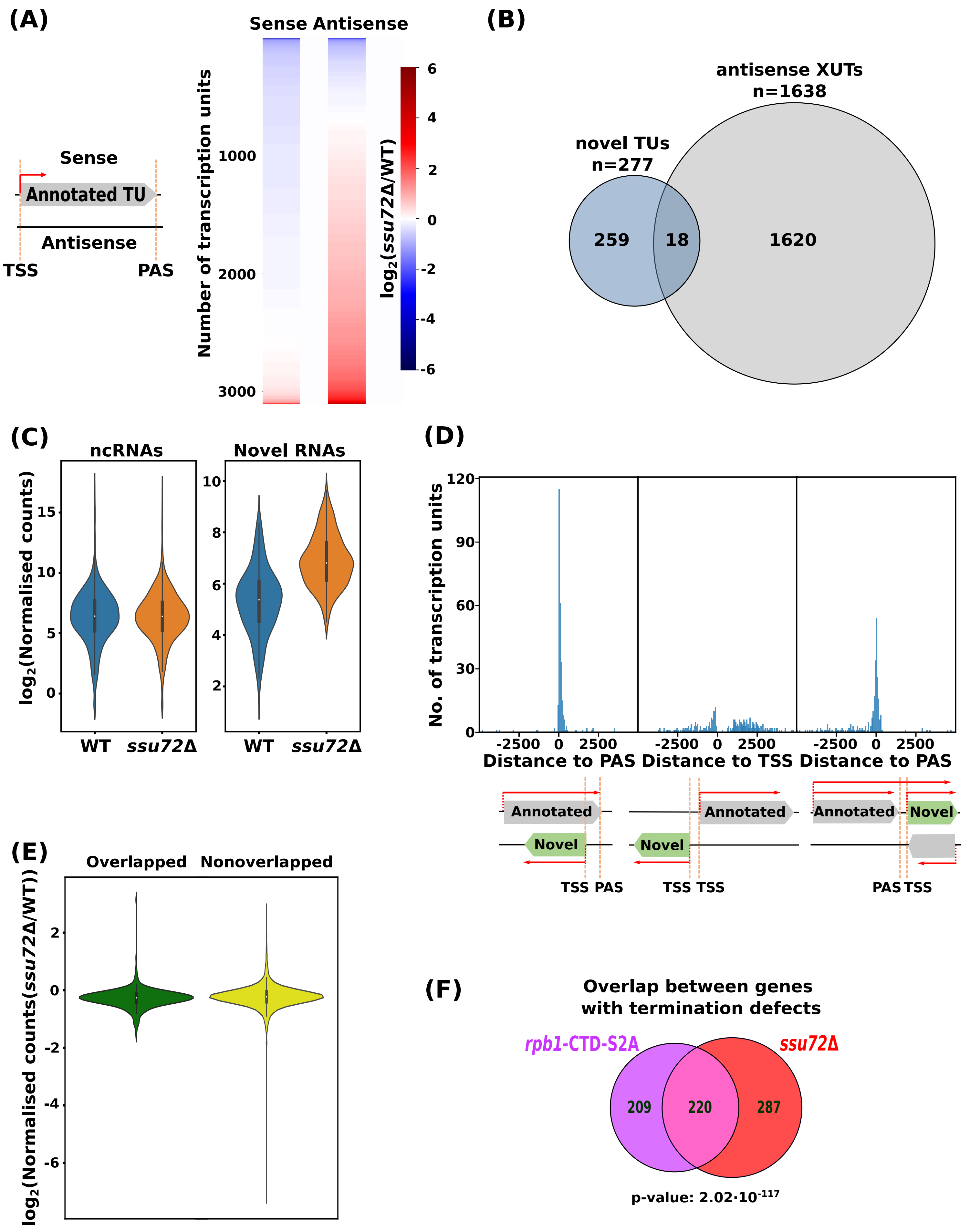

(A)
Sense
Annotated TU
Antisense
TSS
PAS
Sense Antisense
Number of transcription units
1000
2000
3000
log2(ssu72Δ/WT)
6
4
2
0
-2
-4
-6
(B)
antisense XUTs
n=1638
novel TUs
n=277
259
18
1620
(C)
ncRNAs
Novel RNAs
log2(Normalised counts)
WT
ssu72Δ
(D)
No. of transcription units
Distance to PAS
Distance to TSS
Distance to PAS
Annotated
Novel
TSS PAS
TSS TSS
PAS TSS
(E)
Overlapped
Nonoverlapped
log2(Normalised counts(ssu72Δ/WT))
(F)
Overlap between genes
with termination defects
rpb1-CTD-S2A
ssu72Δ
209
220
287
p-value: 2.02·10-117

Figure S2. Deletion of Ssu72 induces cryptic antisense transcripts and readthrough. Related to Figures 4 and 5.
(A) A schematic diagram showing sense strand (coding strand) and antisense strand (template strand) of annotated non-overlapping transcripts units (left panel). Transcripts derived from the coding strand (sense) and the opposite strand (antisense) were sorted based on the fold change (log_2) in the levels in the Ssu72 mutant compared to WT – first batch of experiments (compare Figure 5D, Method section).
(B) Venn diagram showing the overlap between 277 antisense transcripts dependent on Ssu72 and Xrn1–regulated antisense transcripts from (Wery et al., 2018).
(C) Violin plots show changes in the levels of the annotated ncRNAs (left), and novel RNAs (right) in WT and *ssu72*Δ (the spike-in normalised log_2 value – first batch of experiments).
(D) The histogram depicts the distribution of distances from the predicted TSS of novel Ssu72-dependent transcripts (n=277) to the closest PAS and TSS of the annotated TU.
The diagram shows three scenarios: novel transcript starts from the opposite strand within the region close to PAS of the annotated gene (left), novel TSS is closest to the annotated TSS (middle) in the opposite strand, and to annotated PAS (right) on the same strand.
(E) The violin plot analyses assessing changes in the levels of the annotated transcripts overlapping and non-overlapping with antisense transcripts in *ssu72*Δ (log_2 value).
(F) Venn diagram indicating overlap between genes with termination defects in *ssu72*Δ and *rpb1*-CTD-S2A.

Figure S3

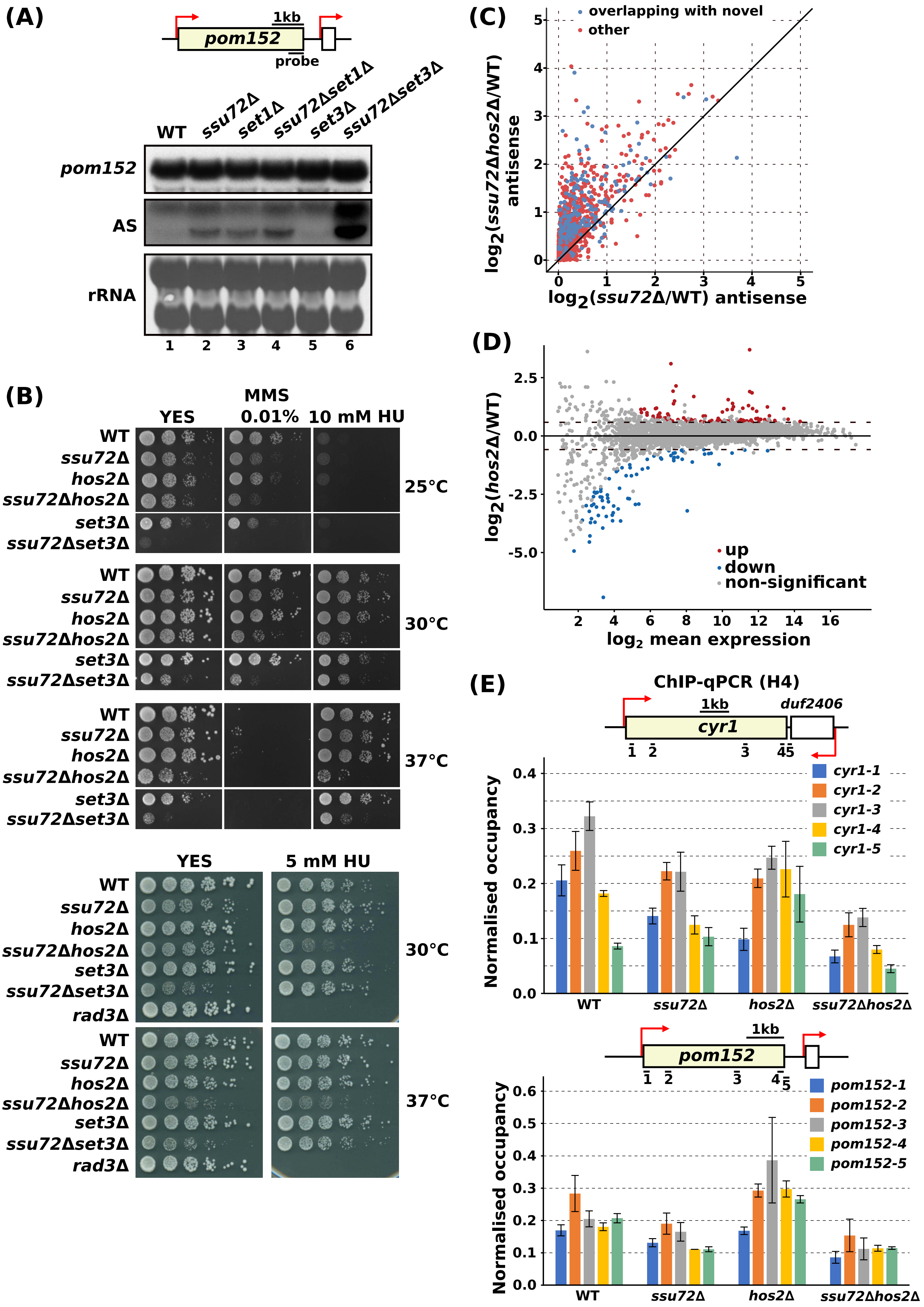
(A)
1kb
pom152
probe
WT
ssu72Δ
set1Δ
ssu72Δset1Δ
set3Δ
ssu72Δset3Δ
pom152
AS
rRNA
1 2 3 4 5 6
(B)
YES
MMS 0.01%
10 mM HU
WT
ssu72Δ
hos2Δ
ssu72Δhos2Δ
set3Δ
ssu72Δset3Δ
25°C
30°C
37°C
YES
5 mM HU
rad3Δ
(C)
overlapping with novel
other
log2(ssu72Δhos2Δ/WT) antisense
log2(ssu72Δ/WT) antisense
(D)
log2(hos2Δ/WT)
log2 mean expression
up
down
non-significant
(E)
ChIP-qPCR (H4)
1kb
duf2406
cyr1
1 2 3 4 5
cyr1-1
cyr1-2
cyr1-3
cyr1-4
cyr1-5
Normalised occupancy
WT
ssu72Δ
hos2Δ
ssu72Δhos2Δ
1kb
pom152
1 2 3 4 5
pom152-1
pom152-2
pom152-3
pom152-4
pom152-5
Normalised occupancy

Figure S3. Hos2 enhances Ssu72-regulated molecular phenotypes. Related to Figures 5 and 6.
(A) Levels of *pom152* mRNA and *pom152* antisense (AS) RNA were analysed by Northern blot in the indicated strains. Methylene blue staining was used to visualize rRNA as a loading control.
(B) Analyses of cellular growth in the presence of genotoxic agents (MMS and HU). The growth of *ssu72*Δ*hos2*Δ is compromised in the presence of HU. Indicated strains (WT, *ssu72*Δ, *hos2*Δ, *set3*Δ, *ssu72*Δ*hos2*Δ and *ssu72*Δ*set3*Δ, *rad3*Δ) were grown at 25°C, 30°C or 37°C temperatures in YES media without or with indicated concentration of drugs.
(C) Hos2 limits Ssu72-dependent antisense transcripts globally. Scatter plot showing the $\log_2$-fold change in the levels of antisense in *ssu72*Δ (x-axis) and *ssu72*Δ *hos2*Δ (y-axis) relative to WT. Each dot represents annotated TU for which antisense relative reads are compared between strains and had a relative value higher than zero. Antisense was separated into two categories: overlapping novel antisense transcripts (n=196 out of 277, blue) and other (n=1466, red). Due lower coverage of the second batch of experiments not all novel antisense transcripts appear as upregulated in *ssu72*Δ. Black diagonal line depicts no change between single and double mutants.
(D) MA plot illustrates transcriptional changes in strain lacking Hos2 compared to WT. Each dot represents annotated transcription unit as described in (Eser et al., 2016), grey reflects no change, red and blue depicts transcripts that were upregulated or downregulated (>1.5 fold, FDR<0.05), respectively. Among upregulated transcripts (94 total) 66 mRNAs were found.
(E) ChIP-qPCR analysis of H4 levels across the *cyr1* and *pom152* gene in WT, *ssu72*Δ, *hos2*Δ and *ssu72*Δ*hos2*Δ. The schematic diagrams show the organisation of *cyr1* or *pom152* loci and positions of primer pairs used for qPCR depicted as back bars. Red arrows indicate the TSS position. The quantification of ChIP-qPCR shows the ratio of IP over the input signal. The error bars represent the standard error of mean (SEM). Results are an average of 3 repeats for WT, *ssu72*Δ and 2 repeats *hos2*Δ, *ssu72*Δ*hos2*Δ, except *pom152* loci where it was repeated twice for all strains.

Figure S4

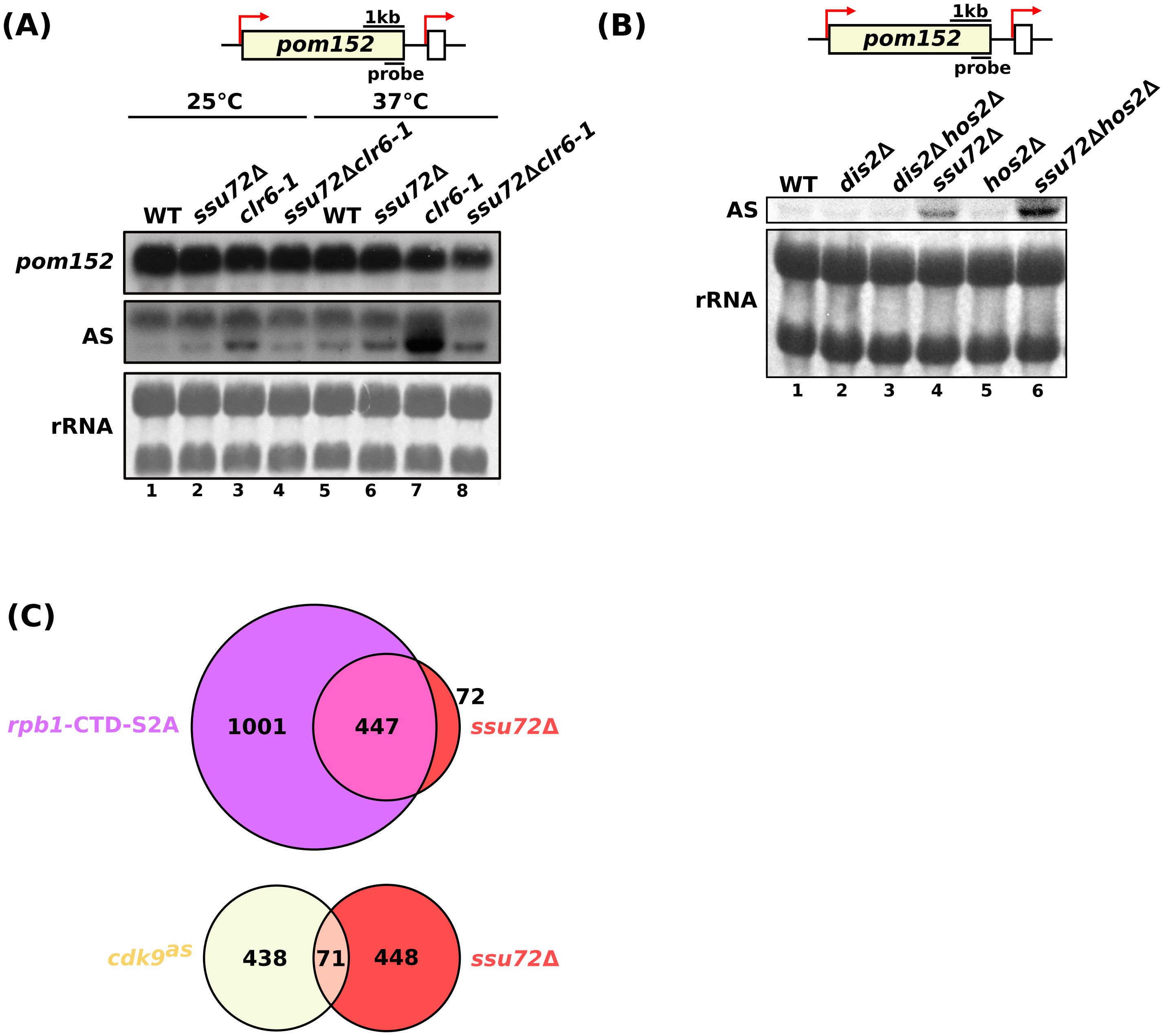

Figure S4. Ssu72 and Rpb1-CTD-S2A mutants accumulate antisense transcripts. Related to Figure 7.
(A) Northern blot analysis for sense and antisense (AS) *pom152* transcripts in indicated strains. To assess RNA levels in temperature-sensitive *clr6-1* strain, RNA was extracted from cells grown at permissive (25°C) and non-permissive (37°C) temperatures. Methylene blue staining was used to visualize rRNA as a loading control.
(B) Northern blot analysis for antisense (AS) *pom152* transcript in indicated strains. Synergistic effect of Hos2 is only associated with the loss of Ssu72 phosphatase. Methylene blue staining was used to visualize rRNA as a loading control.
(C) Venn diagram illustrating the overlap between antisense transcripts dependent on Ssu72, Ser2 phosphorylation of Rpb1 CTD or Cdk9.
