# Supplementary material for: Transcription and chromatin-based surveillance mechanism controls suppression of cryptic antisense transcription
Source: Cell Rep. 2021 Sep 7;36(10):109671. doi: 10.1016/j.celrep.2021.109671 (PMC8441049; doi:10.1016/j.celrep.2021.109671)
Supplement: Document S2. Article plus supplemental information [file mmc9.pdf]

Cell Reports

Article

Transcription and chromatin-based surveillance mechanism controls suppression of cryptic antisense transcription

Graphical abstract

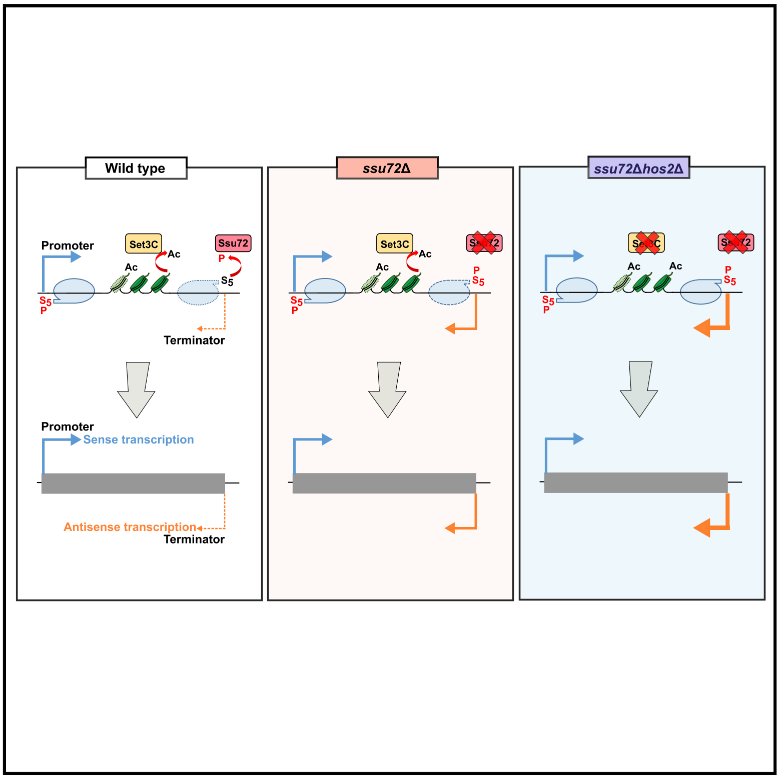

Highlights

- Conserved phosphatase Ssu72 controls RNA polymerase II phosphorylation
- Ssu72 is required for transcription termination at convergent genes
- Antisense transcription is globally increased in the absence of Ssu72
- HDAC Hos2 acts in the absence of Ssu72 to minimize antisense transcription

Authors

Dong-Hyuk Heo, Krzysztof Kuś, Pawel Grzechnik, ..., Soren Nielsen, Nikolay Zenkin, Lidia Vasiljeva

Correspondence

lidia.vasilieva@bioch.ox.ac.uk

In brief

Unwanted antisense transcription might be detrimental for the cell. Heo et al. demonstrate that conserved RNA polymerase II phosphatase Ssu72 and histone deacetylase Hos2 collaborate to keep under control the cryptic antisense transcription that arises from the fission yeast genome.

Heo et al., 2021, Cell Reports *36*, 109671
September 7, 2021 © 2021 The Author(s).
https://doi.org/10.1016/j.celrep.2021.109671

CellPress

Cell Reports

CellPress
OPEN ACCESS

Article

Transcription and chromatin-based surveillance mechanism controls suppression of cryptic antisense transcription

Dong-Hyuk Heo,[1,5] Krzysztof Kuś,[1,5] Pawel Grzechnik,[2] Sue Mei Tan-Wong,[3] Adrien Birot,[1] Tea Kecman,[1] Soren Nielsen,[4] Nikolay Zenkin,[4] and Lidia Vasiljeva[1,6,*]

[1]Department of Biochemistry, University of Oxford, Oxford OX1 3QU, UK
[2]School of Biosciences, College of Life & Environmental Sciences, University of Birmingham, Edgbaston, Birmingham B15 2TT, UK
[3]Sir William Dunn School of Pathology, University of Oxford, South Parks Road, Oxford OX1 3RE, UK
[4]Centre for Bacterial Cell Biology, Biosciences Institute, Faculty of Medical Sciences, Newcastle University, Baddiley-Clark Building, Richardson Road, Newcastle upon Tyne NE2 4AX, UK
[5]These authors contributed equally
[6]Lead contact
*Correspondence: lidia.vasilieva@bioch.ox.ac.uk
https://doi.org/10.1016/j.celrep.2021.109671

SUMMARY

Phosphorylation of the RNA polymerase II C-terminal domain $Y_1S_2P_3T_4S_5P_6S_7$ consensus sequence coordinates key events during transcription, and its deregulation leads to defects in transcription and RNA processing. Here, we report that the histone deacetylase activity of the fission yeast Hos2/Set3 complex plays an important role in suppressing cryptic initiation of antisense transcription when RNA polymerase II phosphorylation is dysregulated due to the loss of Ssu72 phosphatase. Interestingly, although single Hos2 and Set3 mutants have little effect, loss of Hos2 or Set3 combined with *ssu72Δ* results in a synergistic increase in antisense transcription globally and correlates with elevated sensitivity to genotoxic agents. We demonstrate a key role for the Ssu72/Hos2/Set3 mechanism in the suppression of cryptic antisense transcription at the 3′ end of convergent genes that are most susceptible to these defects, ensuring the fidelity of gene expression within dense genomes of simple eukaryotes.

INTRODUCTION

The precise regulation of RNA polymerase II (RNAPII) transcription is important for gene expression, co-transcriptional RNA processing, and chromatin structure (Bae et al., 2020; Bentley, 2014; Ebmeier et al., 2017; Fong et al., 2015; Hsin and Manley, 2012; Muñoz et al., 2010; Nojima et al., 2018; Rosonina et al., 2014; Saldi et al., 2016; Santos-Rosa et al., 2002; Tellier et al., 2020; Vasiljeva et al., 2008; Venkatesh et al., 2012). Recruitment of factors engaged in the regulation of transcription is mediated by phosphorylation of the C-terminal domain (CTD) of the largest subunit of RNAPII. The CTD is modified within a repetitive conserved heptad sequence (Tyr1-Ser2-Pro3-Thr4-Ser5-Pro6-Ser7) (Akhtar et al., 2009; Buratowski, 2009; Corden, 2013; Eick and Geyer, 2013; Fabrega et al., 2003; Govind et al., 2010; Jasnovidova and Stefl, 2013; Ng et al., 2003; Vasiljeva et al., 2008; Zaborowska et al., 2016; Zhang et al., 2012). CTD phosphorylation on Tyr1, Ser2, Thr4, Ser5, and Ser7 is tightly controlled by various kinases and phosphatases (Cho et al., 2001; Harlen and Churchman, 2017; Jeronimo et al., 2016; Kecman et al., 2018a; Lyons et al., 2020; Nemec et al., 2019; Sanso and Fisher, 2013; Tietjen et al., 2010; Yurko and Manley, 2018), establishing RNAPII phosphorylation states specific to each stage of the transcription cycle (referred to as the “CTD code”). Once unphosphorylated RNAPII binds to the promoter, TFIIH unwinds the downstream DNA to form a transcription bubble and phosphorylates Ser5 on the CTD (Holstege et al., 1996; Kim et al., 2000; Komarnitsky et al., 2000). During transcription elongation, Ctk1 and Bur1 kinases phosphorylate Ser2 (Cho et al., 2001; Murray et al., 2001).

Co-transcriptional modifications of chromatin fine-tune transcriptional activity by maintaining repressive chromatin and suppressing deleterious cryptic transcription from promoters and intragenic regions (Carrozza et al., 2005; Joshi and Struhl, 2005; Kim et al., 2012, 2016; Sansó et al., 2020). Like RNAPII phosphorylation, histone modifications occur in a stage-specific manner during transcription. Histone H3 di- and trimethylation on lysine 4 (H3K4me2 and 3) by Set1 takes place early on during transcription, and Set1 recruitment relies on Ser5 phosphorylation of RNAPII (Bae et al., 2020; Kim and Buratowski, 2009; Ng et al., 2003). The deposition of H3K36me during elongation by Set2 is linked to Ser2 phosphorylation (Krogan et al., 2003; Li et al., 2003; Schaft et al., 2003). Furthermore, H3K4me promotes recruitment of the histone deacetylase (HDAC) Hos2 (a part of Set3C complex) to promoters, whereas H3K36me recruits the HDAC Rpd3S to the gene body (Carrozza et al., 2005; Govind et al., 2010; Keogh et al., 2005; Kim and Buratowski, 2009; Kim et al., 2016; Li et al., 2007; Shi et al., 2007; Taverna et al., 2006).

This is an open access article under the CC BY license (http://creativecommons.org/licenses/by/4.0/).

Phosphatases Fcp1 and Ssu72 dephosphorylate Ser2 and Ser5 of RNAPII CTD, respectively (Cho et al., 2001; Krishnamurthy et al., 2004), whereas Thr4-P is removed by protein phosphatase 1 (PP1; or Dis2 in *Schizosaccharomyces pombe*) (Kecman et al., 2018a). In fission yeast, Dis2 (or Glc7 in *Saccharomyces cerevisiae*) and Ssu72 are proposed to interact with the mRNA 3-′end processing machinery (Cleavage Polyadenylation Factor [CPF]) (Casañal et al., 2017; Vanoosthuyse et al., 2014). Other components co-purify with the CPF in fission yeast, namely, scaffold proteins Pta1, Ppn1, and Swd2.2 (homologs to mammalian Symplekin, PNUTS, and WDR82). Ssu72 was shown to interact with the N-terminal part of Symplekin in *S. cerevisiae* and humans (Ghazy et al., 2009; Xiang et al., 2010). PP1 forms complexes with PNUTS and WDR82 in humans (Austenaa et al., 2015, 2021; Lee et al., 2010), suggesting that they might be arranged as a phosphatase module that constitutes part of CPF. A recent study suggested that the PP1-PNUTS-WDR82 complex might form between corresponding fission yeast homologs Dis2-Ppn1-Swd2.2 (Benjamin et al., 2021). PP1, PNUTS, and WDR82 control the expression of non-coding (nc) RNAs produced from enhancer regions (eRNAs) (Austenaa et al., 2015, 2021). Additionally, PP1 was implicated in the transcription termination of nc and protein-coding (pc) genes in yeast and mammals (Cortazar et al., 2019; Eaton et al., 2020; Kecman et al., 2018a; Parua et al., 2018).

Studies in *S. cerevisiae* demonstrated that inactivation of Ssu72 is associated with defective transcription elongation (Dichtl et al., 2002), loss of promoter directionality (Tan-Wong et al., 2012), and transcription termination defects (Kim et al., 2006). Ssu72 acts with the Nrd1-Nab3-Sen1 (NNS) complex in termination of nc transcription independently of the polyadenylation signal (PAS) and with the CPF in mRNA 3′ end processing that relies on PAS (Kim et al., 2006; Steinmetz and Brow, 2003). However, the NNS-mediated transcription termination mechanism is not conserved. Instead, homologs of Nrd1, Seb1, and SCAF4 are involved in a CPF-dependent transcription termination mechanism common to all RNAPII transcripts (pc and nc) in fission yeast and mammals (Gregersen et al., 2019; Larochelle et al., 2018; Lemay et al., 2016; Wittmann et al., 2017). Fission yeast Ssu72 co-purifies with the CPF (Vanoosthuyse et al., 2014), suggesting that it contributes to the CPF function. Indeed, we provide evidence that the loss of Ssu72 leads to an mRNA 3′-end processing and transcription termination defect at a specific subset of genes wherein genes that are in a convergent orientation constitute the majority.

Here, we show that Ssu72 controls CTD phosphorylation and transcription elongation. Upregulated Ser5-P in *ssu72*Δ correlates with a global loss of Ser2-P and increased cryptic antisense transcription. Similarly, the substitution of Ser2 to Ala within all 29 repeats of the CTD results in elongation defects, accumulation of the 3′ extended RNAs at convergent genes, and a global increase in antisense transcription. Unexpectedly, we demonstrate that Ssu72-dependent cryptic antisense transcription is suppressed by Hos2 HDAC, whereas Hos2 alone does not have a noticeable effect on terminator-derived antisense transcription. We propose that Ssu72 and Hos2 act in conjunction to suppress cryptic transcription. Our study reveals a role for chromatin-modifying HDAC activity in the "quality control" of gene expression. We propose a mechanism of how high-fidelity gene expression is achieved within dense genomes of simple eukaryotes.

RESULTS

Loss of Ssu72 leads to a transcription elongation defect in fission yeast

Due to the essential nature of Ssu72 in *S. cerevisiae*, previous studies were limited to the use of temperature-sensitive Ssu72 mutant strains (Pappas and Hampsey, 2000). To further explore the importance of Ssu72 in RNAPII transcription at a physiological temperature, we used a strain lacking Ssu72, as it is nonessential in fission yeast. We assessed the effect on RNAPII levels in the absence of Ssu72 using chromatin immunoprecipitation sequencing (ChIP-seq). Analysis of the spike-in normalized data revealed a reduction in RNAPII occupancy after the first ~250 bp downstream of the transcription start site (TSS) in *ssu72Δ* (Figures 1A and B), suggestive of an elongation defect. Analysis of the RNAPII "traveling ratio" (STAR Methods; Reppas et al., 2006; Shetty et al., 2017) also implies that transcription elongation is compromised in *ssu72Δ* cells (Figures 1C). To further assess the requirement of Ssu72 for normal elongation, we tested whether *ssu72Δ* cells are sensitive to 6-azauracil (6-AU) that reduces RNAPII processivity by depleting the intracellular pool of UTP and GTP (Mason and Struhl, 2005; Shaw and Reines, 2000). The *ssu72Δ* is more sensitive to 6-AU than the wild type (WT) (Figure 1D), which supports the role of Ssu72 in transcription elongation.

Deletion of Ssu72 changes the RNAPII CTD phosphorylation pattern

Ssu72 acts as an RNAPII Ser5 and Ser7 phosphatase in budding yeast and mammals (Krishnamurthy et al., 2004; Xiang et al., 2010, 2012). To assess how Ssu72 affects CTD phosphorylation in fission yeast, we analyzed the distribution of Ser5-P, Ser7-P, Ser2-P, and Tyr1-P (Figures 2 and S1A–S1D) genome wide in WT and *ssu72*Δ cells by using calibrated ChIP-seq. In WT, Ser5-P and Ser7-P peak downstream of the TSS and decrease gradually toward the end of the genes, whereas Ser2-P and Tyr1-P peak at the end of the genes, in agreement with previous reports (Bataille et al., 2012; Mayer et al., 2010). There is a smaller peak of Ser5-P and Ser7-P RNAPII around the PAS region (Figures S1A and S1B). As expected, an increase in Ser5-P and Ser7-P levels was observed in *ssu72*Δ, suggesting that Ssu72 dephosphorylates these residues, which is consistent with data reported for *S. cerevisiae* Ssu72 (Bataille et al., 2012). Surprisingly, we also observed a simultaneous decrease in Ser2-P and Tyr1-P levels, which might explain the reduced elongation rate observed in *ssu72*Δ. Hyper-phosphorylation of Ser5/7 might affect the recruitment or activity of the kinase(s) responsible for Ser2-P/Tyr1-P. In mammalian cells, Tyr1-P was demonstrated to promote phosphorylation on Ser2 (Mayfield et al., 2019), which could explain why we observed a simultaneous decrease of both marks. To test whether the ability of RNAPII to transcribe is directly affected by the lack of functional Ssu72, we purified RNAPII from WT and *ssu72*Δ cells and compared the activity of purified polymerases in an *in vitro* transcription elongation

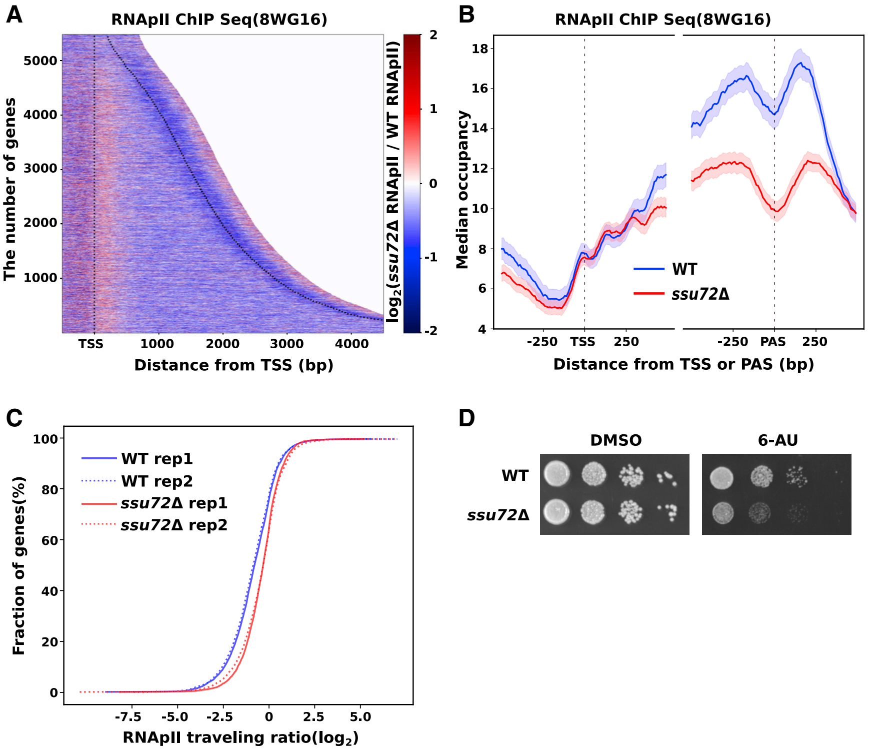

Figure 1. Ssu72 is important for normal transcription elongation

(A) Heatmap shows the distribution of Rpb1 (8WG16) ChIP-seq read counts in 10-bp bins ($\log_2$ (*ssu72*Δ/WT) spike-in normalized to *S. cerevisiae* Rbp1). Each line in the heatmap indicates a TU with an assigned number (n = 5,474) and 500-bp flanking TSS and PAS (dotted lines). All TUs were aligned at TSS and sorted by their lengths (indicated on the y axis). Genes longer than 4.5 kb were trimmed at 4.5 kb.

(B) Metagene profile of Rpb1 median occupancy in WT (blue) and *ssu72Δ* (red). The shaded regions represent 95% confidence intervals.

(C) All genes (n = 5,474) sorted based on the "RNAPII traveling ratio" defined as $\log_2$(5′ occupancy/3′ occupancy) within 200-bp bins in WT (blue) and *ssu72Δ* (red). "The traveling ratio" and corresponding fraction of genes are plotted on the x and y axis, respectively, as a cumulative distribution. Two biological replicates (solid and dotted lines) are shown.

(D) The sensitivity to 6-AU of WT and *ssu72Δ* strains.

See also Figure S1.

assay. RNAPII purified from *ssu72*Δ and WT strains had similar elongation properties (Figures S1E and S1F) supporting the previously suggested role of *trans*-acting factors in mediating the function of RNAPII phosphorylation in transcription.

Dysregulated CTD phosphorylation correlates with altered gene expression in *ssu72Δ*

To assess how transcriptional changes observed in *ssu72*Δ cells affect RNA levels globally, we carried out RNA sequencing (RNA-seq) on rRNA-depleted total RNA isolated from WT and *ssu72*Δ cells. The differential expression analysis revealed that levels of 413 transcripts (316 mRNAs) were significantly decreased (>1.5-fold, $p < 0.05$) (Figure 3A), and those of 132 transcripts (56 mRNAs) were increased in *ssu72*Δ cells (>1.5-fold, $p < 0.05$) (Figure 3A).

Next, we performed a Gene Ontology (GO) analysis of significantly reduced (n = 316) or increased (n = 56) mRNAs. Genes encoding for components of cellular biosynthesis pathways were downregulated in *ssu72*Δ cells ($p = 4.1 \cdot 10^{-19}$; Table S1). In contrast, levels of mRNAs encoding proteins involved in iron uptake were increased (Figures 3B and 3C). This is in line with a study reporting upregulation of mRNAs encoding proteins involved in iron metabolism in a catalytically defective Ssu72 mutant (Sanchez et al., 2019). Interestingly, a similar effect on iron-responsive genes was reported for RNAPII CTD Y1F or S2A RNAPII CTD mutants (Schwer et al., 2014). Therefore, dysregulated CTD phosphorylation on Ser2 and Tyr1 in *ssu72Δ* cells may contribute to an increased expression of mRNAs engaged in iron uptake.

Loss of Ssu72 leads to the induction of cryptic antisense transcription

Mutations in transcription elongation factors were shown to lead to increased initiation of transcription at cryptic sites (Cheung et al., 2008; DeGennaro et al., 2013; Kaplan et al., 2003; Shetty et al., 2017). Therefore, we wanted to test whether the elongation defect observed in *ssu72*Δ cells coincides with increased cryptic transcription.

A mild, global decrease in the number of reads along the gene body of annotated transcription units (TUs) was observed, which is in agreement with the elongation defect in *ssu72*Δ cells (Figure 4A). On the other hand, an increase in the number of reads produced from the opposite strand was observed in *ssu72*Δ (Figure 4B). We selected only non-overlapping TUs (3,107 out of 5,474; Eser et al., 2016) for subsequent analysis. We then assessed the changes in the expression level of transcripts produced from the coding strand of the annotated TUs as well as from the opposite strand in *ssu72Δ* (Figure S2A). This assessment revealed that the expression of 267 transcripts (218 mRNAs) was decreased, whereas the expression of 51 transcripts (39 mRNAs) was increased (>1.5-fold, $p < 0.05$) upon loss of Ssu72. In contrast, an increase in antisense reads for 519 TUs was observed in *ssu72Δ* cells. We evaluated whether concordantly mapped antisense reads induced in the *ssu72Δ* mutant can be assembled into continuous transcripts. We have identified 277 potential novel nc transcripts predicted to be longer than 50 nucleotides (nt) and upregulated by the deletion of Ssu72 (>1.5 fold; Table S2; as described in STAR Methods). A total of 265 out of the predicted 277 transcripts have overlapping TUs on the opposite strand. Previous work demonstrated that cryptic antisense transcription occurring in yeast genomes (*S. cerevisiae* and *S. pombe*) can yield unstable transcripts degraded by the exonuclease Xrn1 (named XUTs) (van Dijk et al., 2011; Wery et al., 2018). However, levels

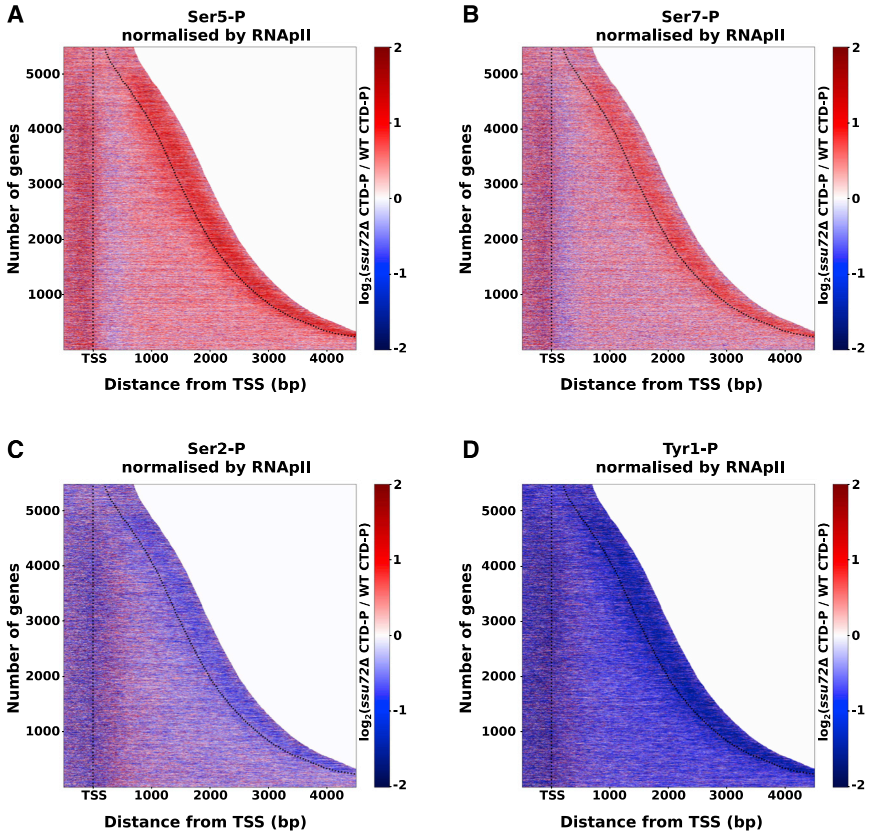

Figure 2. The deletion of Ssu72 causes significant changes in RNAPII CTD phosphorylation

Heatmap shows $\log_2$ ratios between *ssu72Δ* and WT of CTD phosphorylation on Ser5 (A), Ser7 (B), Ser2 (C), and Tyr1 (D) using 10-bp bins. Data were normalized by the level of Rpb1 shown in Figure 1. Each line in the heatmap indicates a TU with an assigned number.

See also Figure S1.

of only a small number of XUTs (18 out of 1,638 XUTs) are increased in the absence of Ssu72 (Figure S2B). To examine whether depletion of Ssu72 also leads to increased expression of other previously annotated nc transcripts, we analyzed how their abundance is affected in the *ssu72Δ*. The abundance of the annotated ncRNAs is not significantly changed in *ssu72Δ* cells, implying that Ssu72 is responsible for the derepression of a specific subset of the cryptic transcripts (Figure S2C).

It was shown that gene promoters or terminators can give rise to antisense transcription (van Dijk et al., 2011; Neil et al., 2009; Tan-Wong et al., 2012; Xu et al., 2009). Ssu72 was proposed to suppress antisense transcription derived from the bi-directional promoters in *S. cerevisiae* (Tan-Wong et al., 2012), but it is not clear whether promoter bi-directionality is a prominent feature in fission yeast. To assess whether Ssu72 regulates antisense transcription arising from promoters or terminators, we measured the distance between the predicted TSS of these transcripts and the closest annotated PAS and TSS on the same or the opposite strands (Figure S2D). The TSS of the Ssu72-dependent transcripts aligns well with the position of annotated PAS on both strands but not with the TSS of annotated genes (Figure S2D). Interestingly, 223 antisense transcripts were produced from the loci where annotated genes are organized in convergent orientation (Figure S2D), such as *cyr1-spbc19c7.04c* (Figures 5A and 5B). Because RNA-seq cannot reliably map the TSS of novel transcripts seen in *ssu72Δ*, novel antisense transcripts could result either from the new transcription initiation event at the terminators of the annotated genes or the read-through transcription from the upstream convergent genes (Figure S2D).

Although the biological consequences of pervasive or cryptic transcription are not fully understood, it is known that overlapping antisense transcription could affect the transcription of coding genes on the opposite DNA strand (Berretta et al., 2008; Camblong et al., 2007; Castelnuovo et al., 2013; Wery et al., 2018). To investigate how Ssu72-dependent antisense transcription correlates with changes in the expression of overlapping TUs, we split known genes into two groups, namely, those that overlap with the novel antisense transcripts (n = 241) and those that do not (n = 2,866), and compared normalized counts for these two groups. This investigation revealed a reduced abundance of annotated transcripts in the Ssu72 mutant regardless of cryptic transcription (Figure S2E). We conclude that an increase in antisense transcription does not have a significant effect on the steady-state levels of overlapping pc transcripts.

Ssu72 contributes to the transcription termination of selected genes

Although no obvious increase in RNAPII occupancy downstream of the PAS is seen globally (Figures 1A and 1B), we analyzed whether individual genes show a defect in 3′ end processing and transcription termination in the absence of Ssu72. First, we analyzed RNA-seq data to find genes that show an increase in the reads downstream of the annotated PAS, which is observed when mRNA 3′ end processing is impaired (Wittmann et al., 2017). To be able to correlate RNA-seq data with the RNAPII profile, we selected TUs that do not have another gene on the same strand (within 250 bp upstream and downstream) for analysis (4,870 out of 6,952 TUs using combined annotation from Eser et al., 2016 and Lock et al., 2019). We identified 507 TUs (441 pc genes) with more than a 2-fold increase in reads past the PAS (Table S3; Figures 4C and 4D). Accumulation of the 3′ read-through RNAs coincides with the shift in RNAPII occupancy in these regions in *ssu72Δ* (Figure 4D). We conclude that Ssu72 contributes to 3′ end processing and transcription termination of selected genes in fission yeast. We noticed that 358 out of 507 TUs with 3′ read-through (~70%) were in a convergent orientation

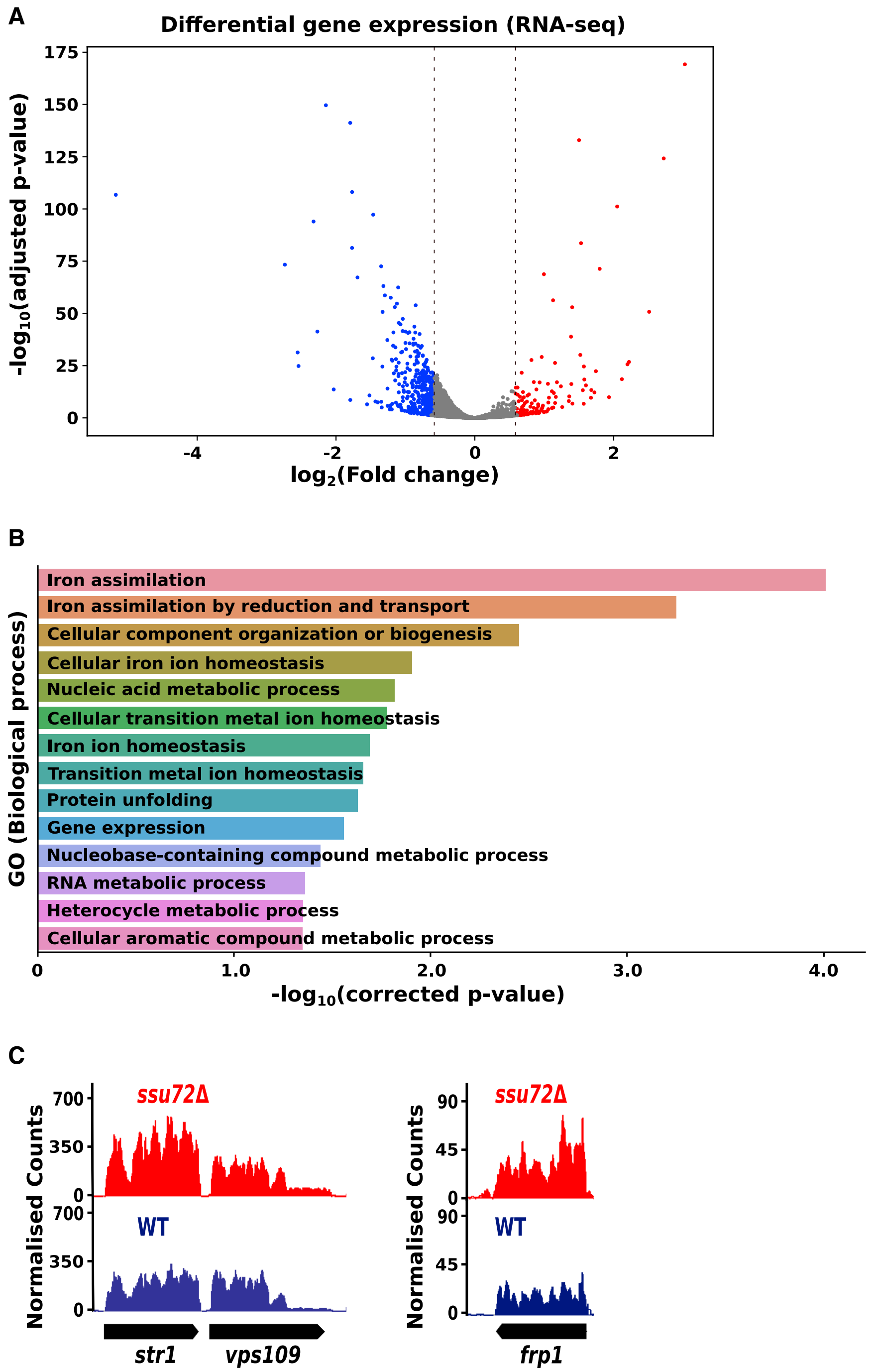

Figure 3. Loss of Ssu72 causes changes in the transcriptome

(A) A volcano plot shows significantly up- (in red) and downregulated (in blue) genes in *ssu72Δ* (p < 0.05, >1.5-fold).

(B) Gene Ontology of the transcripts upregulated in *ssu72*Δ.

(C) RNA-seq genome browser tracks for iron-responsive genes *str1* and *frp1* from WT (blue) and *ssu72*Δ (red).

See also Table S1.

to the neighboring gene. This finding is consistent with a study that reported a transcription termination defect at convergent genes in the absence of Swd2.2, another component of the proposed phosphatase module (Vanoosthuyse et al., 2014). To determine whether the read-through transcription overlaps with the antisense transcription observed within convergent loci in *ssu72*Δ, we analyzed whether the genes located upstream of the 277 novel antisense transcripts show 3′ read-through. Indeed, 51 out of the 507 genes defective in termination were found upstream of novel antisense transcripts, suggesting that 51 out of 277 antisense RNAs are derived from read-through transcription rather than *de novo* transcription initiation. We conclude that Ssu72 suppresses promiscuous transcription either through ensuring accurate termination or preventing unwanted transcription initiation at the convergent regions. We asked whether defective 3′ end processing of mRNA observed in the *ssu72Δ* mutant could be due to the loss of Ser2-P in this strain. To assess the contribution of Ser2-P, we performed RNA-seq with an S2A mutant of RNAPII that revealed accumulation of the 3′ extended RNA species (Figures 4C and S2F). Interestingly, more than one-half of the genes affected also accumulated read-through RNAs in the absence of Ssu72, suggesting that decreased phosphorylation on Ser2 could underpin the transcription termination defect observed in the Ssu72 mutant (Figure S2F). This result is in agreement with the study showing defective recruitment of the mRNA 3′−end processing factors when *S. cerevisiae* Ser2-P kinase Ctk1 is deleted (Ahn et al., 2004).

Set3C/Hos2-mediated surveillance mechanism prevents the activation of cryptic transcription in the absence of Ssu72

HDAC complexes were implicated in preventing unwanted cryptic transcription in *S. cerevisiae*. Thus, Set2/Rpd3S and Set1/Set3/Hos2 complexes are involved in suppressing transcription initiation from cryptic promoters within the gene body and 5′ regions (Carrozza et al., 2005; Keogh et al., 2005; Kim and Buratowski, 2009; Li et al., 2007). In fission yeast, Hos2 and Clr6 are homologs of *S. cerevisiae* Hos2 and Rpd3, respectively (Wang et al., 2002; Wirén et al., 2005). We, therefore, tested whether HDACs are involved in the regulation of cryptic transcripts suppressed by Ssu72. Although most

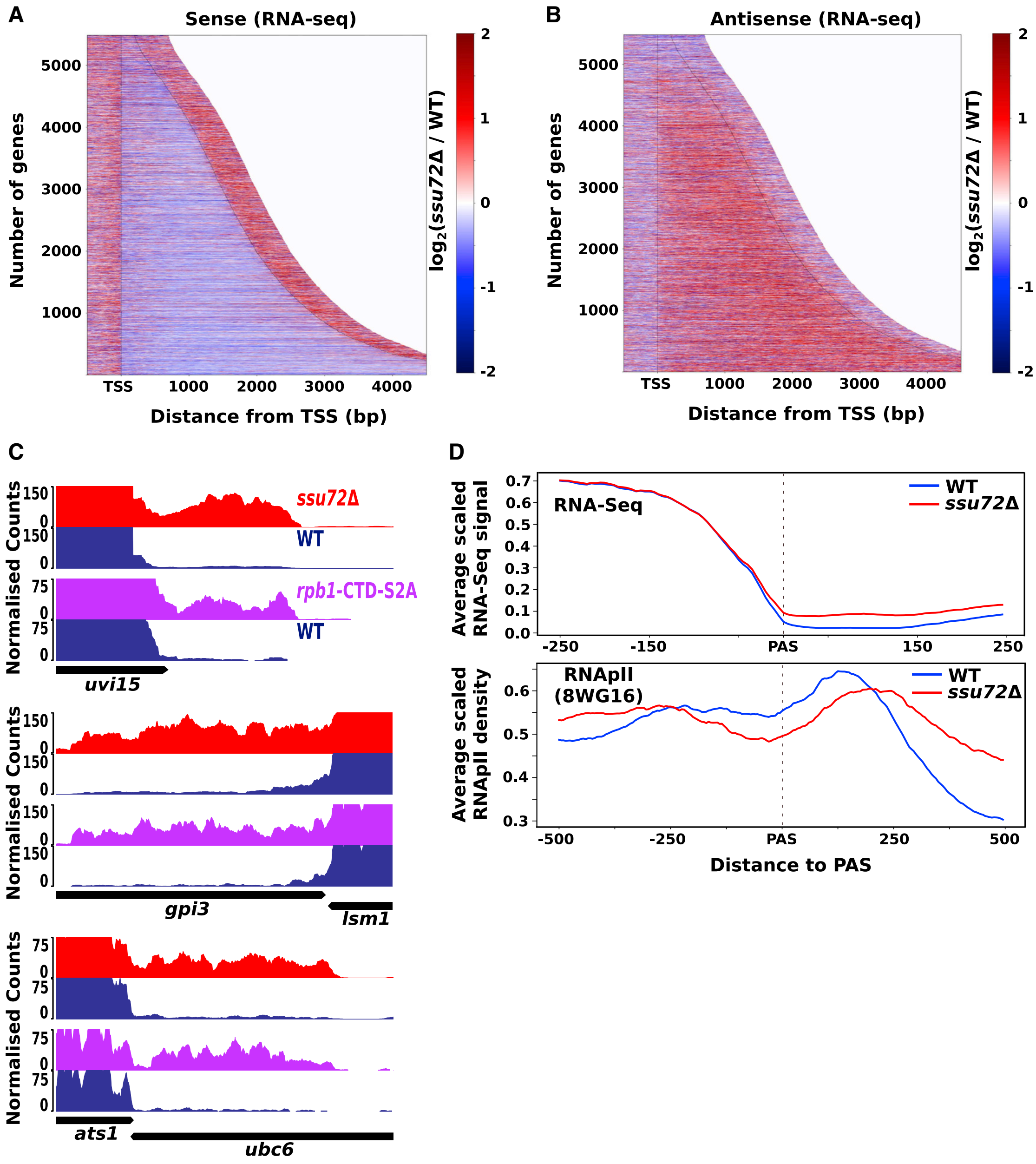

Figure 4. Cells lacking Ssu72 show increased antisense (AS) transcription

(A and B) Heatmaps showing the log_2 ratio value of the number of reads (*ssu72*Δ/WT) mapped to the sense (A) and the AS (B) strands. Each line indicates a TU. All TUs were aligned at TSS and sorted by length.

(C) RNA-seq genome browser tracks depicting 3′-end read-through transcription in *ssu72*Δ and *rpb1*-CTD-S2A. The tracks of WT, *ssu72*Δ, and *rpb1*-CTD-S2A are blue, red, and violet, respectively.

(D) Metagene profiles of RNA-seq and RNAPII ChIP data show defective 3′-end processing and RNAPII termination at selected genes (n = 507) in *ssu72Δ*. See also Figure S2 and Tables S2 and S3.

genomic loci in which we detected longer Ssu72-dependent antisense transcripts are represented by convergent genes, such as *cyr1-spbc19c7.04c*, some cases are not convergent, such as *pom152-spncRNA.1527* (Figure 5A) that were selected as two representative loci for subsequent analysis. In agreement with RNA-seq results, the accumulation of cryptic antisense transcripts produced from *pom152* and *cyr1* was observed in *ssu72Δ* cells (Figure 5B). In contrast, antisense transcription was not observed in the Hos2 single mutant (Figure 5B). Strikingly, a strong synergistic accumulation of antisense RNA was observed in the *hos2Δssu72Δ* compared with the single *ssu72Δ* mutant (Figure 5B, lanes 2, 4, 6, and 8) suggesting that in the context of functional phosphorylation of RNAPII, HDAC activity is not required and Ssu72 alone is sufficient to repress cryptic antisense transcription.

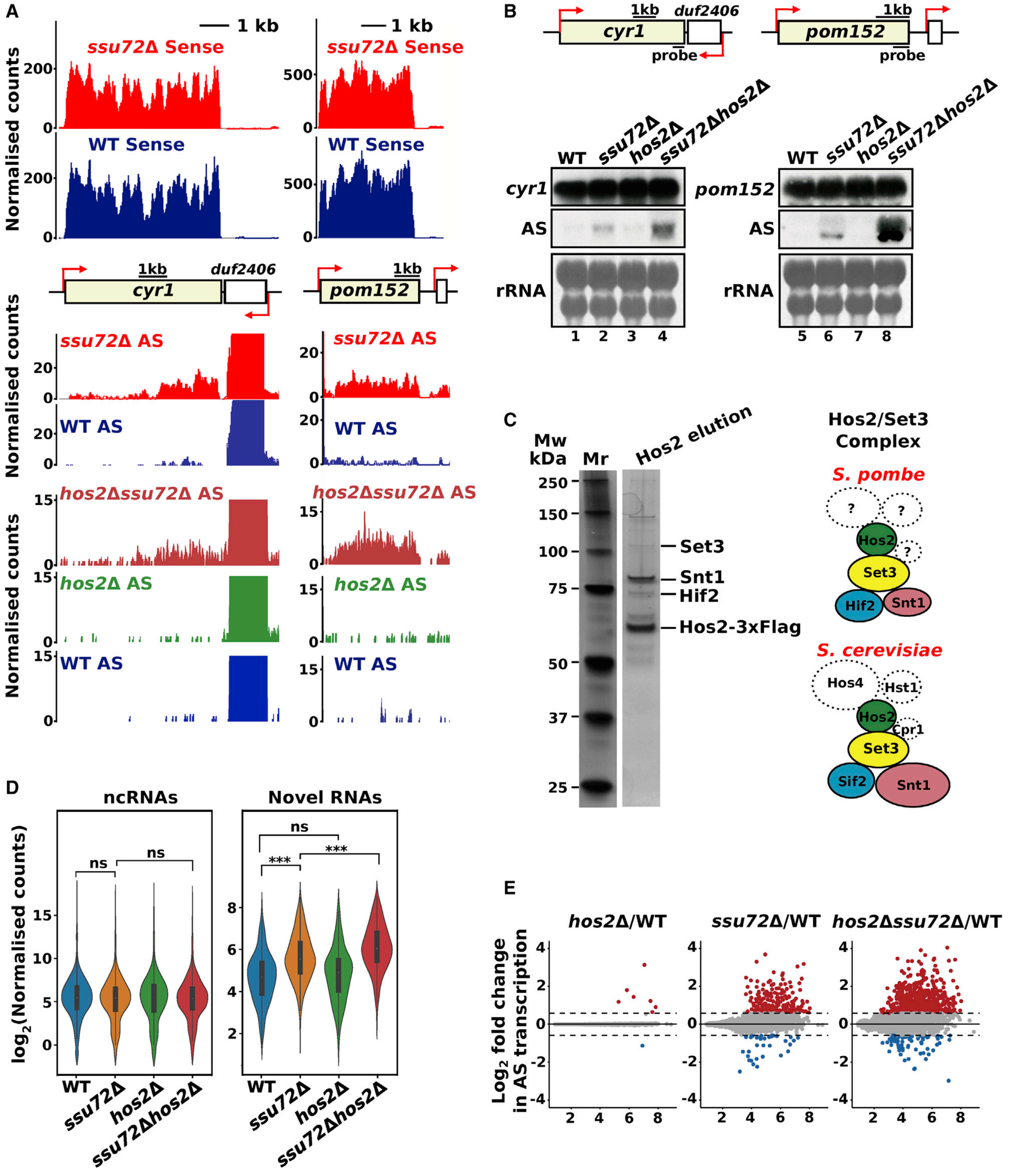

Figure 5. The Hos2 HDAC complex represses cryptic AS transcription in the absence of Ssu72

(A) RNA-seq genome browser tracks for representative loci (*cyr1* and *pom152*) and their neighboring genes (*spbc19c7.04c* and *spncrna.1527*). The tracks of WT, *ssu72*Δ, *hos2*Δ, and *hos2*Δ*ssu72*Δ are blue, red, green, and crimson, respectively. For each gene, seven tracks are presented, as follows: the first four are derived from the first batch of experiments and the remaining from the second batch (compare Figures 5D and 5E).

(B) Levels of the AS RNA and mRNA produced from the annotated genes (*cyr1* and *pom152*) were assessed by northern blot in the indicated strains.

(legend continued on next page)

Hos2 was proposed to be a catalytic subunit of the Set3 complex (Set3C) containing Set3, Snt1, Sif2, Hos4, Hst1, and Cpr1 proteins in *S. cerevisiae* (Pijnappel et al., 2001). To identify components of the *S. pombe* Set3C, we purified the native Hos2 complex by using affinity and size exclusion chromatography followed by mass spectrometry analysis (Table S4). Purification has revealed that Set3, Snt1, and Hif2 (a homolog of *S. cerevisiae* Sif2) proteins form a stable complex with Hos2 (Figure 5C). Another HDAC, Hst1, is a part of Set3C in *S. cerevisiae* (Pijnappel et al., 2001). However, our data suggest that, in fission yeast, Hos2 is the only HDAC associated with Set3C. Next, we tested whether an entire complex or only Hos2 is required for suppression of antisense transcription. Similarly to *ssu72*Δ*hos2*Δ, *ssu72*Δ*set3*Δ cells show synergistic derepression of cryptic transcription (Figure S3A, lanes 2, 5, and 6). We conclude that Set3C plays a specific role in the suppression of cryptic transcription in the absence of Ssu72, representing a chromatin-based surveillance mechanism. The functional synergy between Ssu72 and the Hos2/Set3C HDAC complex is also evident from the effect of simultaneous deletion of Ssu72 and Set3C on growth (Figure S3B). A strain lacking both Ssu72 and Set3 grows noticeably slower than the WT and each of the single mutants (*ssu72*Δ and *set3*Δ). Interestingly, the synergistic growth defect observed in *ssu72*Δ*set3*Δ is further exacerbated when cells are grown on plates containing agents that induce DNA damage or interfere with DNA replication, such as methyl methanesulfonate (MMS) and hydroxyurea (HU). A similar sensitivity is also seen in *ssu72*Δ*hos2*Δ. Additive sensitivity to elevated and low temperatures is also observed in these mutants, suggesting that severe derepression of cryptic transcription might affect proper stress responses.

To investigate whether Hos2 is required to prevent transcription at cryptic promoters genome wide, we performed RNA-seq in WT, *ssu72*Δ, *hos2*Δ, and *ssu72*Δ*hos2*Δ cells (Figure 5D). Interestingly, the *hos2*Δ single mutant does not show a significant change of cryptic transcription within 277 genomic loci where we find Ssu72 regulated transcripts (the median of $\log_2$-normalized counts [MLNC] = 4.9 in *hos2*Δ) compared with the WT (MLNC = 4.7). In contrast, the *ssu72*Δ*hos2*Δ mutant (MLNC = 6) shows a significant increase in the levels of cryptic transcripts compared with WT and a single *ssu72*Δ mutant (MLNC = 5.5). A similar tendency is observed globally; only a few antisense transcripts were detected in a Hos2 single mutant compared with unperturbed cells (Figure 5E), whereas levels of multiple antisense transcripts are increased in *ssu72*Δ. A strong additive increase in the abundance and number of the antisense transcripts is observed in *ssu72*Δ*hos2*Δ compared to those of single *ssu72*Δ (Figures 5E and S3C). This finding suggests that Hos2 plays a key role in suppressing antisense transcription genome wide upon loss of functional Ssu72 when phosphorylation of the RNAPII is dysregulated.

In agreement with Hos2 acting as an HDAC, an increase in H4 acetylation is observed in *hos2Δ* and *ssu72Δhos2Δ* at *cyr1*, *pom152*, and *rps401* genes (Figure 6A) with the most prominent effect seen at the promoter. We have assessed whether the loss of Hos2 affects the expression of these genes. Only selected mRNAs are affected by Hos2 depletion (where 66 mRNAs are increased >1.5 fold, $p < 0.05$), including those involved in stress response, sporulation, cell cycle control, and iron assimilation (Figure S3D; Table S5).

We noticed lower levels of H4 in *ssu72Δ* than those in the WT, which are even further reduced in *ssu72Δhos2Δ* at the loci associated with antisense transcription (*cyr1* and *pom152*) (Figure S3E). Additionally, terminators of genes with antisense transcription show lower nucleosome occupancy than randomly selected genes (Figure 6B). This result suggests that a lower nucleosome occupancy might render these loci more susceptible to the loss of Ssu72 and favor antisense transcription.

Previous studies demonstrated that HDAC complex Clr6 (Rpd3S in *S. cerevisiae*) is involved in the suppression of intragenic antisense transcription (Nicolas et al., 2007; Sansó et al., 2020). Accordingly, we observed a strong increase in antisense transcription in *clr6-1* (Figure S4A, lanes 3 and 7). The deletion of Ssu72 leads to a partial suppression of antisense transcription in *clr6-1* (Figure S4A, lanes 4 and 8), suggesting that the increased antisense transcription observed in *ssu72*Δ is not due to compromised Clr6 function. Instead, functional Ssu72 appears to be required for antisense transcription observed in the Clr6 mutant. It is possible that in the absence of Ssu72, Hos2 can suppress Clr6-dependent antisense transcripts. Although outside the scope of the current work, it would be interesting to test this possibility in the future.

To further explore functional links between Ssu72 and chromatin modifiers, we have tested the contribution of H3K4 methyltransferase Set1 that was implicated in the recruitment of the Set3C complex to promoter regions. In contrast to the *ssu72*Δ*hos2*Δ, the *ssu72*Δ*set1*Δ mutant did not show a synergistic increase in antisense transcription (Figure S3A, lanes 1–4), although some levels of the antisense RNA were detectable in each single mutant.

Characterization of the loci associated with the Ssu72-dependent antisense transcription

To further understand how Ssu72 represses antisense transcription, we asked whether transcripts detected in *ssu72*Δ can be initiated from the terminator region by examining whether general

(C) Purification of the Hos2 HDAC complex. Gel-filtration elution fractions containing Hos2 were analyzed by silver-stained SDS-PAGE and mass spectrometry. The diagram depicts the Hos2/Set3 complexes in *S. cerevisiae* (bottom) and *S. pombe* (top).
(D) The violin plot of levels of novel AS transcripts and previously annotated ncRNA ($\log_2$ value spike-in normalized reads) in indicated strains. These plots are based on the second batch of experiments; compare Figures 5A and 5E and STAR Methods section. The significance is calculated according to the Mann-Whitney U test.
(E) Hos2 deletion enhances AS transcription in the absence of Ssu72. MA plots of shrunk $\log_2$ fold changes illustrate changes in the AS production in indicated strains. Each dot represents annotated non-overlapped TU for which AS reads were compared between strains; gray reflects no change, and red and blue depict AS transcripts that were upregulated or downregulated (>1.5 fold), respectively. These analyses are based on the second batch of experiments (WT, *ssu72*Δ, *hos2*Δ, and *ssu2*Δ*hos2*Δ; refer to STAR Methods section).
See also Figures S2 and S3 and Tables S4 and S5.

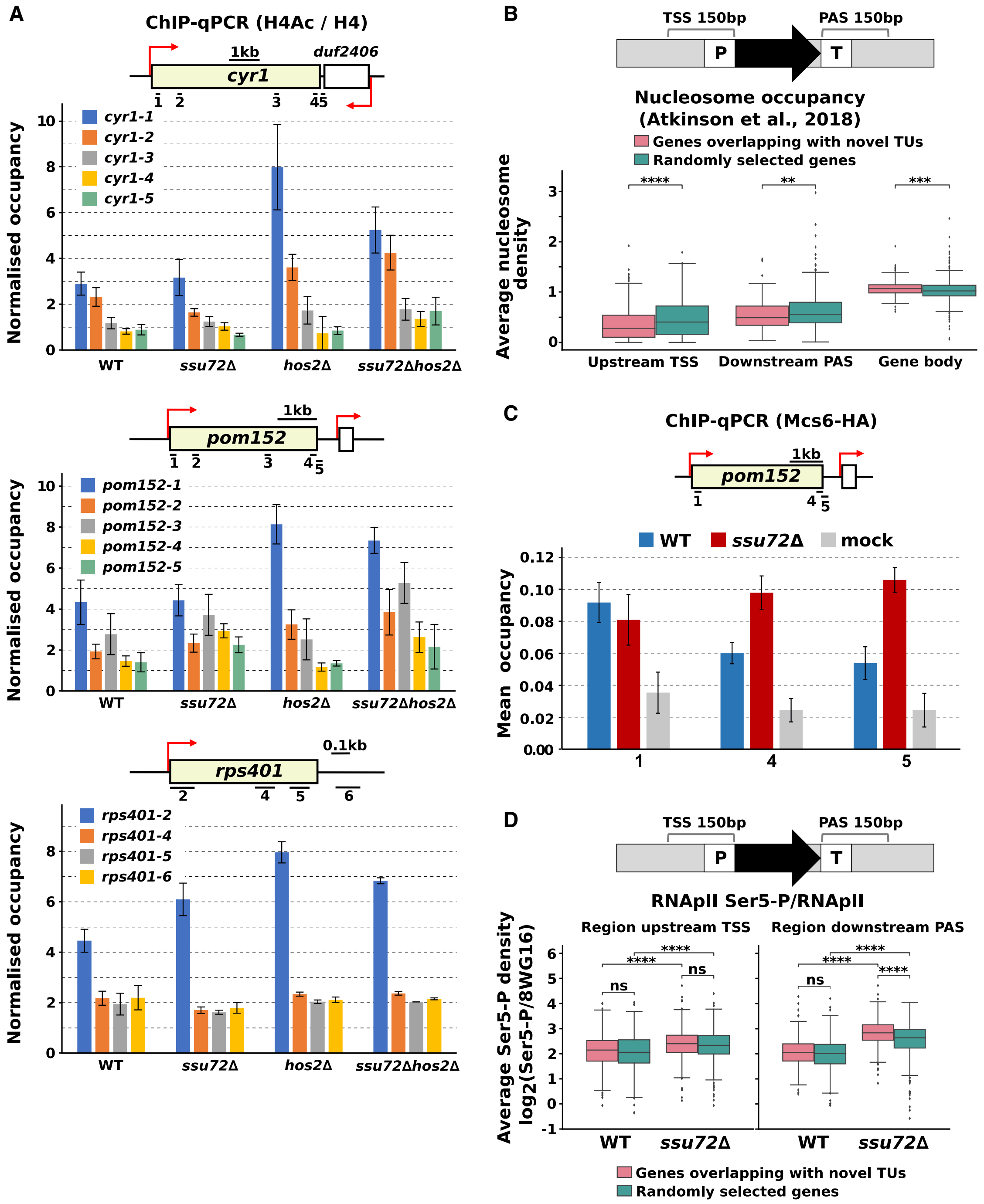

Figure 6. Characterization of the Ssu72-dependent AS transcripts

(A) ChIP-qPCR analysis of relative H4 acetylation (H4Ac/H4) levels across the *cyr1*, *pom152*, and *rps401* genes in WT, *ssu72*Δ, *hos2*Δ, and *ssu72*Δ*hos2*Δ. The diagram shows the organization of loci and positions of primer pairs used for qPCR depicted as back bars. Red arrows indicate TSS position. The quantification of ChIP-qPCR shows the ratio of IP over the input signal. Error bars represent the standard error of the mean (SEM). Results are an average of 3 repeats for WT and *ssu72*Δ and 2 repeats for *hos2*Δ and *ssu72*Δ*hos2*Δ, except *pom152* loci were repeated twice for all strains.

(B) The presence of AS transcription *ssu72*Δ is associated with decreased nucleosome occupancy at the TSS and PAS. Boxplots showing nucleosome occupancy over 150 nt of the TSS and PAS of genes with Ssu72-dependent AS transcripts (277 TUs) (pink) and randomly selected 554 genes (green) by using nucleosome occupancy data from Atkinson et al. (2018). The significance is calculated either according to the Mann-Whitney U test for independent or Wilcoxon rank-sum test for matching gene sets. Multiple testing was controlled by Bonferroni correction.

(legend continued on next page)

transcription initiation factors are recruited to the terminator region in *ssu72Δ*. Recruitment of the kinase component of TFIIH, Mcs6 (Cdk7 in mammals), that phosphorylates RNAPII CTD on Ser5 was assessed in *ssu72Δ* and WT strains (Figure 6C). This showed increased Mcs6 occupancy at the terminator region of *pom152*. Although the Mcs6 signal was not high, possibly due to transient association of the kinase during transcription, the change observed in *ssu72Δ* is reproducible. Consistent with this finding, an increase in Ser5-P levels was more prominent at the terminators of the genes with antisense transcription than those at terminators of the random genes (Figure 6D). In contrast to *ssu72Δ*, deletion of the phosphatase Dis2, involved in mediating efficient transcription termination in fission yeast and mammalian cells (Cortazar et al., 2019; Kecman et al., 2018a; Parua et al., 2018), had no effect on the accumulation of the *pom152* antisense RNA (Figure S4B) either on its own or in combination with Hos2 deletion. These data suggest that at least in some instances, antisense transcription could be a result of *de novo* transcription initiation rather than read-through from the upstream gene.

We assessed how the loss of Ser2-P on RNAPII in the S2A mutant affected the accumulation of antisense transcription. Like Ssu72 mutants, levels of multiple antisense transcripts were significantly increased (n = 1,448, >1.5 fold, false discovery rate [FDR] < 0.05) in the S2A mutant (Figures 7A, 7B, and S4C). Most of the Ssu72-dependent antisense transcripts also accumulated in the S2A mutant (447 out of 519), suggesting that decreased Ser2-P might contribute to the increase in antisense transcription in the absence of Ssu72 (Figure S4C). As in the Ssu72 mutant, a global reduction of RNA levels was observed in the RNAPII S2A mutant, possibly due to defective transcription elongation (Figure 7C). It was reported that inhibition of the catalytic activity of an analog sensitive mutant of Cdk9 kinase results in derepression of antisense transcription produced from the gene bodies of selected genes in fission yeast (Sansó et al., 2020). Furthermore, the Cdk9-affected group of genes was enriched for genes with promoter-proximal pausing without any bias toward convergent gene pairs. We observed no difference in RNAPII levels upstream of promoters for Ssu72-dependent genes compared to randomly selected genes (Figure 7D; Booth et al., 2016), implying that Ssu72 and Cdk9 affect cryptic transcription of different groups of genes. Indeed, only a minor fraction of the antisense transcripts observed upon Cdk9 inhibition were increased in the Ssu72 mutant (Figure S4C). Our observations that the loss of Ssu72 mimics the S2A phenotype is in line with a previous study that identified Lsk1 as a major kinase responsible for Ser2-P in fission yeast (Coudreuse et al., 2010).

DISCUSSION

Post-translational modifications on histones and RNAPII play important roles in gene regulation. We reveal a molecular link between these two processes by demonstrating that the HDAC complex Set3C acts in the absence of RNAPII phosphatase Ssu72 to suppress the accumulation of cryptic antisense transcription (Figure 7E).

The loss of Ssu72 results in a global decrease in transcription elongation but increased initiation of cryptic transcription from terminators and transcriptional read-through. The latter defects are particularly pronounced at convergent genes. A previous study also proposed a role for Ssu72 in the suppression of antisense transcription in *S. cerevisiae*, where it regulates antisense transcription at promoters rather than terminators (Tan-Wong et al., 2012). This implies that Ssu72 has a conserved, although mechanistically distinct function, in the suppression of cryptic transcription in different yeast species. Our data suggest that upregulation of cryptic transcription at convergent genes may arise from new initiation events or a failure to terminate transcription when RNAPII phosphorylation is dysregulated in *ssu72Δ*. Interestingly, loci associated with cryptic antisense transcription in the Ssu72 mutant show lower nucleosome density. This finding is consistent with the observation that convergent genes show high occupancy of the histone variant H2A.Z (that has a preference for nucleosome-free regions). It was also demonstrated that convergent genes are more prone to antisense transcription even in unperturbed cells (Ni et al., 2010). The Set3C/Hos2 complex was implicated in mediating repositioning and remodeling of H2A.Z containing nucleosomes (Hang and Smith, 2011), which might explain why convergent genes are primarily affected by the Ssu72/Hos2 pathway. On the other hand, reduced nucleosome occupancy at the 3′ end of the genes can also impair the efficiency of transcription termination and facilitate transcriptional read-through (Hildreth et al., 2020). We notice that RNAPII phosphorylation on Ser5 is affected more prominently at gene terminators associated with Ssu72-dependent antisense transcription, which may explain why these regions are more sensitive to the loss of Ssu72.

We demonstrate that deletion of the HDAC subunit Hos2 of the Set3C does not lead to the induction of antisense transcription or show a major effect on the steady-state levels of mRNAs genome wide in fission yeast. Consistently, in *S. cerevisiae*, Hos2/Set3C contributes to the regulation of a specific subset of pc genes involved in DNA damage response (Sharma et al., 2007), sporulation (Pijnappel et al., 2001), and nc transcription that overlaps metabolic genes mediating the kinetics of their induction (Kim et al., 2012; Wang et al., 2002). However, deletion of Hos2 leads to a striking additive increase in antisense transcription in the cells lacking Ssu72. We demonstrate that Set3C suppresses antisense transcription from gene terminators derived from either *de novo* transcription initiation or read-through from the downstream gene. This suggests that the role of chromatin modifiers in suppressing nc transcription is not limited to the promoter and gene body. However, because the effect on H4 acetylation in the absence of Hos2 outside of promoter regions is

(C) Msc6-HA ChIP-qPCR. Positions of primer pairs used for qPCR are depicted as back bars. The quantification of ChIP-qPCR shows the ratio of IP over input relative to WT. The error bars represent SEM. Experiments were repeated 3 times except for the mock (non-tagged strain, n = 2).
(D) Boxplots indicating changes in RNAPII Ser5-P/total RNAPII (8WG16) in *ssu72Δ* over 150 nt of the TSS and PAS of genes with Ssu72-dependent AS transcripts (277 TUs) (pink) and randomly selected 554 genes (green). Statistical analysis as in (B).
See also Figure S3.

CellPress
OPEN ACCESS

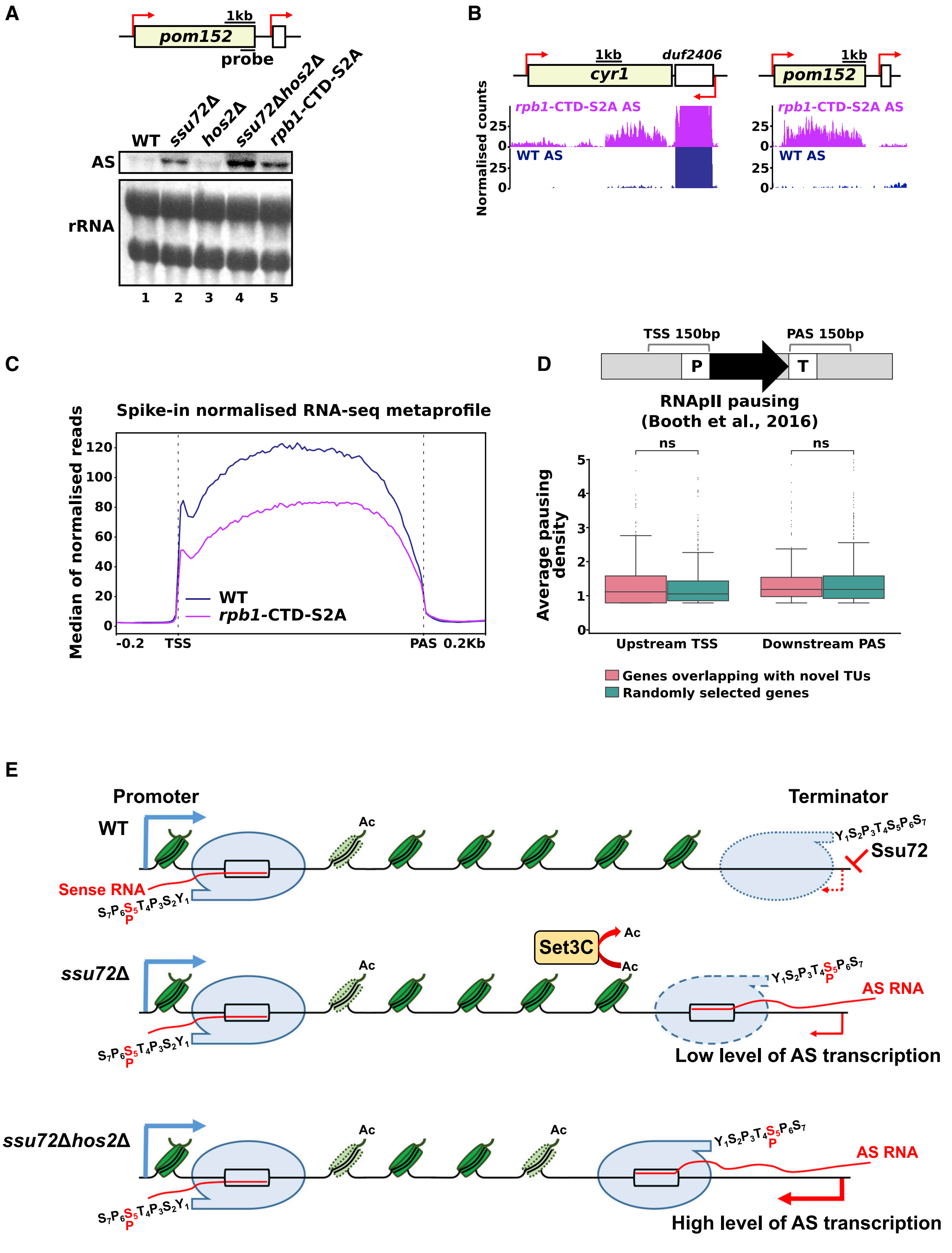

Figure 7. Loss of RNAPII Ser2 phosphorylation correlates with the accumulation of AS transcription and elongation defect

(A) Levels of the AS RNA produced from *pom152* were assessed by northern blot in the indicated strains. rRNA is used as a loading control.

(B) RNA-seq genome browser tracks presenting AS transcription at representative loci (*cyr1* and *pom152*) and their neighboring genes (*spbc19c7.04c* and *spncrna.1527*). The tracks of WT and *rpb1*-CTD-S2A are blue and violet, respectively.

(C) Metaprofile of RNA-seq data in WT (blue) and RNAPII-S2A strains (violet). The median of spike-in normalized reads is plotted for genes that do not have any annotated transcript on the same strand in the 250-bp region either upstream of the TSS or downstream of the PAS.

(D) RNAPII pausing at TSS or PAS in *ssu72*Δ for groups of genes as in (B) by using RNAPII pausing data from Booth et al. (2016). Statistical analysis as in Figure 6B.

(legend continued on next page)

rather mild, we do not rule out the possibility that Hos2 could also mediate the repression of antisense transcription by deacetylating other substrates or independently of its catalytic activity. It is also possible that increased antisense transcription in the Ssu72 mutant renders terminators more sensitive to alterations in the chromatin structure upon the loss of Set3C, leading to additive induction of nc transcription.

Co-transcriptional recruitment of Set3C, Rpd3L, and Rpd3S to promoters and gene body regions relies on histone methylation by Set1 and Set2 (Carrozza et al., 2005; Govind et al., 2010; Keogh et al., 2005; Kim and Buratowski, 2009; Kim et al., 2016; Li et al., 2007; Shi et al., 2007). Previous studies demonstrated that Set1 is recruited to promoters by recognizing Ser5-phosphorylated RNAPII (Materne et al., 2015). In contrast, Ser2 phosphorylation was proposed to prevent Set1 recruitment. Depletion of Ssu72 is accompanied by a global change in the RNAPII phosphorylation profile. This is manifested in the establishment of the "promoter-like" RNAPII phosphorylation pattern at terminator regions, namely, increased Ser5 and decreased Ser2 phosphorylation, which might facilitate Set3C function at terminators. Indeed, an increase in the levels of Ssu72-dependent ncRNAs in an RNAPII S2A mutant lacking Ser2 phosphorylation supports this model. Surprisingly, Set1 and Set3C appear to act independently in the repression of cryptic transcription at terminators. Set3C is only required for the repression of terminator-derived antisense transcription when RNAPII phosphorylation is dysregulated. In contrast, Set1 contributes to the repression of antisense transcription at the *pom152* locus in the presence of Ssu72 and simultaneous deletion of Set1 and Ssu72 does not show synergistic accumulation of antisense RNAs. However, even though Set3C can also be recruited independently of Set1 (Govind et al., 2010), Set3C does not fully repress antisense transcription, suggesting that despite the "promoter-like" profile of RNAPII phosphorylation observed in the absence of Ssu72, terminators are not as efficient as promoters in suppressing antisense transcription, and other factors in addition to Ser5-P might contribute to mediating efficient recruitment of Hos2 at promotors.

Why does cryptic transcription need to be minimized? Cryptic transcription can affect the expression of the neighboring genes (Berretta et al., 2008; Camblong et al., 2009; Kaikkonen and Adelman, 2018; Shah et al., 2014; Watts et al., 2018). Cryptic transcription does not always affect steady-state levels of RNA expressed from the overlapping gene but can modulate the gene induction kinetics in response to environmental changes (Kim et al., 2012; Venkatesh et al., 2016). This raises an interesting possibility that Ssu72-dependent nc transcription may contribute to gene regulation under specific conditions, although it does not appear to affect the steady-state levels of the overlapping pc transcripts. Accumulation of ncRNA in the nucleus was proposed to lead to increased formation of R-loop structures (RNA:DNA hybrids) caused by nascent RNA hybridizing to upstream DNA behind elongating RNAPII (Gavaldá et al., 2013; Nojima et al., 2018; Pefanis et al., 2014, 2015; Santos-Pereira and Aguilera, 2015; Skourti-Stathaki et al., 2014) and rendering cells sensitive to genotoxic drugs (Santos-Pereira et al., 2013). Increased pervasive nc transcription could also interfere with DNA replication (Candelli et al., 2018; Soudet et al., 2018). This might explain why cells that show elevated levels of nc transcription due to a lack of both Ssu72 and HDAC Set3C show synthetic growth defects in the presence of genotoxic and DNA-replication-blocking drugs. Future studies are required to understand a potential link between cryptic transcription and sensitivity to drugs observed in these mutants.

STAR★METHODS

Detailed methods are provided in the online version of this paper and include the following:

- KEY RESOURCES TABLE
- RESOURCE AVAILABILITY
  - Lead contact
  - Materials availability
  - Data and code availability
- EXPERIMENTAL MODEL AND SUBJECT DETAILS
  - Yeast strains
- METHOD DETAILS
  - Yeast techniques
  - Chromatin immunoprecipitation (ChIP-Seq and ChIP-qPCR)
  - ChIP-Seq data analysis
  - RNA sequencing (RNA-Seq)
  - RNA-Seq data analysis
  - Identification of novel transcripts
  - Identification of genes with potential 3′ read-through transcription
  - Northern blot
  - Hos2 purification
  - *In vitro* transcription
- QUANTIFICATION AND STATISTICAL ANALYSIS

SUPPLEMENTAL INFORMATION

Supplemental information can be found online at https://doi.org/10.1016/j.celrep.2021.109671.

ACKNOWLEDGMENTS

We thank Naomi Petela, Ewa Chrostek, Caspar Kengeter, and members of the Vasiljeva lab for the critical reading of the manuscript. We thank the National BioResource Yeast Project, Damien Hermand, Beate Schwer, and Matthew Whitby for the yeast strains. This work was supported by a Wellcome Trust

(E) Model depicting proposed mechanism for Ssu72 and Hos2/Set3C in the suppression of the cryptic transcription. Loss of Ssu72 leads to increased levels of Ser5-P RNAPII at the terminator regions. The decreased amount of RNAPII reaching the terminator might lead to an increase in the initiation of the AS transcription (as suggested by the Msc6 ChIP). For simplicity, the model depicts only RNAPIIs at the promoter (for sense orientation) and terminator (for AS orientation). Loss of Ssu72 and Hos3/Set3C leads to an additive increase in AS transcription possibly due to a combined effect of decreased RNAPII elongation at terminators and altered acetylation of Hos2 substrates.
See also Figure S4.

CellPress
OPEN ACCESS

Senior Research fellowship to L.V. (WT106994/Z/15/Z), a fellowship from the Korean National Research Foundation (NRF) by the Ministry of Education (2014R1A6A3A03060067) to D.-H.H., Sir Henry Dale Fellowship jointly funded by the Wellcome Trust and the Royal Society to P.G. (200473/Z/16/Z), and Wellcome Trust Investigator Awards to N.Z. (102851/Z/13/Z and 217189/Z/19/Z).

AUTHOR CONTRIBUTIONS

D.-H.H., K.K., and L.V. conceived and designed the experiments and wrote the manuscript. D.-H.H. carried out ChIP-seq, ChIP-qPCR, RNA-seq, Northern blot, protein purification, initial bioinformatics analysis, and plate spotting assay. K.K. performed strains construction, protein purifications, and most of the bioinformatics analysis. P.G. performed most of the ChIP-qPCR analysis. A.B. contributed to strain construction and yeast growth assay, T.K. helped with ChIP-seq. S.M.T.-W. performed DRIP and qPCR. S.N. performed the *in vitro* transcription assay in the N.Z. laboratory. All authors edited the manuscript.

DECLARATION OF INTERESTS

The authors declare no competing interests.

Received: February 25, 2020
Revised: August 26, 2020
Accepted: August 13, 2021
Published: September 7, 2021

SUPPORTING CITATIONS

The following references appear in the supplemental information: Grewal et al. (2012); Lemieux et al. (2011); Rentas et al. (2012); Schwer et al. (2012).

REFERENCES

Ahn, S.H., Kim, M., and Buratowski, S. (2004). Phosphorylation of serine 2 within the RNA polymerase II C-terminal domain couples transcription and 3′ end processing. Mol. Cell *13*, 67–76.

Akhtar, M.S., Heidemann, M., Tietjen, J.R., Zhang, D.W., Chapman, R.D., Eick, D., and Ansari, A.Z. (2009). TFIIH kinase places bivalent marks on the carboxy-terminal domain of RNA polymerase II. Mol. Cell *34*, 387–393.

Atkinson, S.R., Marguerat, S., Bitton, D.A., Rodríguez-López, M., Rallis, C., Lemay, J.F., Cotobal, C., Malecki, M., Smialowski, P., Mata, J., et al. (2018). Long noncoding RNA repertoire and targeting by nuclear exosome, cytoplasmic exonuclease, and RNAi in fission yeast. RNA *24*, 1195–1213.

Austenaa, L.M.I., Barozzi, I., Simonatto, M., Masella, S., Della Chiara, G., Ghisletti, S., Curina, A., de Wit, E., Bouwman, B.A.M., de Pretis, S., et al. (2015). Transcription of Mammalian cis-Regulatory Elements Is Restrained by Actively Enforced Early Termination. Mol. Cell *60*, 460–474.

Austenaa, L.M.I., Piccolo, V., Russo, M., Prosperini, E., Polletti, S., Polizzese, D., Ghisletti, S., Barozzi, I., Diaferia, G.R., and Natoli, G. (2021). A first exon termination checkpoint preferentially suppresses extragenic transcription. Nat. Struct. Mol. Biol. *28*, 337–346.

Bae, H.J., Dubarry, M., Jeon, J., Soares, L.M., Dargemont, C., Kim, J., Geli, V., and Buratowski, S. (2020). The Set1 N-terminal domain and Swd2 interact with RNA polymerase II CTD to recruit COMPASS. Nat. Commun. *11*, 2181.

Bähler, J., Wu, J.Q., Longtine, M.S., Shah, N.G., McKenzie, A., 3rd, Steever, A.B., Wach, A., Philippsen, P., and Pringle, J.R. (1998). Heterologous modules for efficient and versatile PCR-based gene targeting in Schizosaccharomyces pombe. Yeast *14*, 943–951.

Bataille, A.R., Jeronimo, C., Jacques, P.É., Laramée, L., Fortin, M.È., Forest, A., Bergeron, M., Hanes, S.D., and Robert, F. (2012). A universal RNA polymerase II CTD cycle is orchestrated by complex interplays between kinase, phosphatase, and isomerase enzymes along genes. Mol. Cell *45*, 158–170.

Benjamin, B., Sanchez, A.M., Garg, A., Schwer, B., and Shuman, S. (2021). Structure-function analysis of fission yeast cleavage and polyadenylation factor (CPF) subunit Ppn1 and its interactions with Dis2 and Swd22. PLoS Genet. *17*, e1009452.

Bentley, D.L. (2014). Coupling mRNA processing with transcription in time and space. Nat. Rev. Genet. *15*, 163–175.

Berretta, J., Pinskaya, M., and Morillon, A. (2008). A cryptic unstable transcript mediates transcriptional trans-silencing of the Ty1 retrotransposon in S. cerevisiae. Genes Dev. *22*, 615–626.

Bitton, D.A., Schubert, F., Dey, S., Okoniewski, M., Smith, G.C., Khadayate, S., Pancaldi, V., Wood, V., and Bähler, J. (2015). AnGeLi: A tool for the analysis of gene lists from fission yeast. Front. Genet. *6*, 330.

Bolger, A.M., Lohse, M., and Usadel, B. (2014). Trimmomatic: a flexible trimmer for Illumina sequence data. Bioinformatics *30*, 2114–2120.

Booth, G.T., Wang, I.X., Cheung, V.G., and Lis, J.T. (2016). Divergence of a conserved elongation factor and transcription regulation in budding and fission yeast. Genome Res. *26*, 799–811.

Buratowski, S. (2009). Progression through the RNA polymerase II CTD cycle. Mol. Cell *36*, 541–546.

Camblong, J., Iglesias, N., Fickentscher, C., Dieppois, G., and Stutz, F. (2007). Antisense RNA stabilization induces transcriptional gene silencing via histone deacetylation in S. cerevisiae. Cell *131*, 706–717.

Camblong, J., Beyrouthy, N., Guffanti, E., Schlaepfer, G., Steinmetz, L.M., and Stutz, F. (2009). Trans-acting antisense RNAs mediate transcriptional gene cosuppression in S. cerevisiae. Genes Dev. *23*, 1534–1545.

Candelli, T., Challal, D., Briand, J.B., Boulay, J., Porrua, O., Colin, J., and Libri, D. (2018). High-resolution transcription maps reveal the widespread impact of roadblock termination in yeast. EMBO J. *37*, e97490.

Carrozza, M.J., Li, B., Florens, L., Suganuma, T., Swanson, S.K., Lee, K.K., Shia, W.J., Anderson, S., Yates, J., Washburn, M.P., and Workman, J.L. (2005). Histone H3 methylation by Set2 directs deacetylation of coding regions by Rpd3S to suppress spurious intragenic transcription. Cell *123*, 581–592.

Casañal, A., Kumar, A., Hill, C.H., Easter, A.D., Emsley, P., Degliesposti, G., Gordiyenko, Y., Santhanam, B., Wolf, J., Wiederhold, K., et al. (2017). Architecture of eukaryotic mRNA 3′-end processing machinery. Science *358*, 1056–1059.

Castelnuovo, M., Rahman, S., Guffanti, E., Infantino, V., Stutz, F., and Zenklusen, D. (2013). Bimodal expression of PHO84 is modulated by early termination of antisense transcription. Nat. Struct. Mol. Biol. *20*, 851–858.

Chen, S., Zhou, Y., Chen, Y., and Gu, J. (2018). fastp: an ultra-fast all-in-one FASTQ preprocessor. Bioinformatics *34*, i884–i890.

Cheung, V., Chua, G., Batada, N.N., Landry, C.R., Michnick, S.W., Hughes, T.R., and Winston, F. (2008). Chromatin- and transcription-related factors repress transcription from within coding regions throughout the Saccharomyces cerevisiae genome. PLoS Biol. *6*, e277.

Cho, E.J., Kobor, M.S., Kim, M., Greenblatt, J., and Buratowski, S. (2001). Opposing effects of Ctk1 kinase and Fcp1 phosphatase at Ser 2 of the RNA polymerase II C-terminal domain. Genes Dev. *15*, 3319–3329.

Corden, J.L. (2013). RNA polymerase II C-terminal domain: Tethering transcription to transcript and template. Chem. Rev. *113*, 8423–8455.

Cortazar, M.A., Sheridan, R.M., Erickson, B., Fong, N., Glover-Cutter, K., Brannan, K., and Bentley, D.L. (2019). Control of RNA Pol II Speed by PNUTS-PP1 and Spt5 Dephosphorylation Facilitates Termination by a "Sitting Duck Torpedo" Mechanism. Mol. Cell *76*, 896–908.e4.

Coudreuse, D., van Bakel, H., Dewez, M., Soutourina, J., Parnell, T., Vandenhaute, J., Cairns, B., Werner, M., and Hermand, D. (2010). A gene-specific requirement of RNA polymerase II CTD phosphorylation for sexual differentiation in S. pombe. Curr. Biol. *20*, 1053–1064.

DeGennaro, C.M., Alver, B.H., Marguerat, S., Stepanova, E., Davis, C.P., Bähler, J., Park, P.J., and Winston, F. (2013). Spt6 regulates intragenic and antisense transcription, nucleosome positioning, and histone modifications genome-wide in fission yeast. Mol. Cell. Biol. *33*, 4779–4792.

Dichtl, B., Blank, D., Ohnacker, M., Friedlein, A., Roeder, D., Langen, H., and Keller, W. (2002). A role for SSU72 in balancing RNA polymerase II transcription elongation and termination. Mol. Cell *10*, 1139–1150.

Dobin, A., Davis, C.A., Schlesinger, F., Drenkow, J., Zaleski, C., Jha, S., Batut, P., Chaisson, M., and Gingeras, T.R. (2013). STAR: ultrafast universal RNA-seq aligner. Bioinformatics *29*, 15–21.

Eaton, J.D., Francis, L., Davidson, L., and West, S. (2020). A unified allosteric/torpedo mechanism for transcriptional termination on human protein-coding genes. Genes Dev. *34*, 132–145.

Ebmeier, C.C., Erickson, B., Allen, B.L., Allen, M.A., Kim, H., Fong, N., Jacobsen, J.R., Liang, K., Shilatifard, A., Dowell, R.D., et al. (2017). Human TFIIH Kinase CDK7 Regulates Transcription-Associated Chromatin Modifications. Cell Rep. *20*, 1173–1186.

Eick, D., and Geyer, M. (2013). The RNA polymerase II carboxy-terminal domain (CTD) code. Chem. Rev. *113*, 8456–8490.

Eser, P., Wachutka, L., Maier, K.C., Demel, C., Boroni, M., Iyer, S., Cramer, P., and Gagneur, J. (2016). Determinants of RNA metabolism in the Schizosaccharomyces pombe genome. Mol. Syst. Biol. *12*, 857.

Fabrega, C., Shen, V., Shuman, S., and Lima, C.D. (2003). Structure of an mRNA capping enzyme bound to the phosphorylated carboxy-terminal domain of RNA polymerase II. Mol. Cell *11*, 1549–1561.

Fong, N., Brannan, K., Erickson, B., Kim, H., Cortazar, M.A., Sheridan, R.M., Nguyen, T., Karp, S., and Bentley, D.L. (2015). Effects of Transcription Elongation Rate and Xrn2 Exonuclease Activity on RNA Polymerase II Termination Suggest Widespread Kinetic Competition. Mol. Cell *60*, 256–267.

Forsburg, S.L. (2003). S. pombe strain maintenance and media. Curr. Protoc. Mol. Biol. *Chapter 13*, Unit 13.15.

Gavaldá, S., Gallardo, M., Luna, R., and Aguilera, A. (2013). R-loop mediated transcription-associated recombination in trf4Δ mutants reveals new links between RNA surveillance and genome integrity. PLoS One *8*, e65541.

Ghazy, M.A., He, X., Singh, B.N., Hampsey, M., and Moore, C. (2009). The essential N terminus of the Pta1 scaffold protein is required for snoRNA transcription termination and Ssu72 function but is dispensable for pre-mRNA 3′-end processing. Mol. Cell. Biol. *29*, 2296–2307.

Govind, C.K., Qiu, H., Ginsburg, D.S., Ruan, C., Hofmeyer, K., Hu, C., Swaminathan, V., Workman, J.L., Li, B., and Hinnebusch, A.G. (2010). Phosphorylated Pol II CTD recruits multiple HDACs, including Rpd3C(S), for methylation-dependent deacetylation of ORF nucleosomes. Mol. Cell *39*, 234–246.

Gregersen, L.H., Mitter, R., Ugalde, A.P., Nojima, T., Proudfoot, N.J., Agami, R., Stewart, A., and Svejstrup, J.Q. (2019). SCAF4 and SCAF8, mRNA Anti-Terminator Proteins. Cell *177*, 1797–1813.e18.

Grewal, C., Hickmott, J., Rentas, S., and Karagiannis, J. (2012). A conserved histone deacetylase with a role in the regulation of cytokinesis in Schizosaccharomyces pombe. Cell Div. *7*, 13.

Hang, M., and Smith, M.M. (2011). Genetic analysis implicates the Set3/Hos2 histone deacetylase in the deposition and remodeling of nucleosomes containing H2A.Z. Genetics *187*, 1053–1066.

Harlen, K.M., and Churchman, L.S. (2017). The code and beyond: transcription regulation by the RNA polymerase II carboxy-terminal domain. Nat. Rev. Mol. Cell Biol. *18*, 263–273.

Hildreth, A.E., Ellison, M.A., Francette, A.M., Seraly, J.M., Lotka, L.M., and Arndt, K.M. (2020). The nucleosome DNA entry-exit site is important for transcription termination and prevention of pervasive transcription. eLife *9*, 1–31.

Holstege, F.C.P., van der Vliet, P.C., and Timmers, H.T.M. (1996). Opening of an RNA polymerase II promoter occurs in two distinct steps and requires the basal transcription factors IIE and IIH. EMBO J. *15*, 1666–1677.

Hsin, J.P., and Manley, J.L. (2012). The RNA polymerase II CTD coordinates transcription and RNA processing. Genes Dev. *26*, 2119–2137.

Jasnovidova, O., and Stefl, R. (2013). The CTD code of RNA polymerase II: a structural view. Wiley Interdiscip. Rev. RNA *4*, 1–16.

Jeronimo, C., Langelier, M.F., Bataille, A.R., Pascal, J.M., Pugh, B.F., and Robert, F. (2016). Tail and Kinase Modules Differently Regulate Core Mediator Recruitment and Function In Vivo. Mol. Cell *64*, 455–466.

Joshi, A.A., and Struhl, K. (2005). Eaf3 chromodomain interaction with methylated H3-K36 links histone deacetylation to Pol II elongation. Mol. Cell *20*, 971–978.

Kaikkonen, M.U., and Adelman, K. (2018). Emerging Roles of Non-Coding RNA Transcription. Trends Biochem. Sci. *43*, 654–667.

Kaplan, C.D., Laprade, L., and Winston, F. (2003). Transcription elongation factors repress transcription initiation from cryptic sites. Science *301*, 1096–1099.

Kecman, T., Kuś, K., Heo, D.H., Duckett, K., Birot, A., Liberatori, S., Mohammed, S., Geis-Asteggiante, L., Robinson, C.V., and Vasiljeva, L. (2018a). Elongation/Termination Factor Exchange Mediated by PP1 Phosphatase Orchestrates Transcription Termination. Cell Rep. *25*, 259–269.e5.

Kecman, T., Heo, D.H., and Vasiljeva, L. (2018b). Profiling RNA Polymerase II Phosphorylation Genome-Wide in Fission Yeast. In Methods in Enzymology, A.J. Carpousis, ed. (Academic Press), pp. 489–504.

Keogh, M.C., Kurdistani, S.K., Morris, S.A., Ahn, S.H., Podolny, V., Collins, S.R., Schuldiner, M., Chin, K., Punna, T., Thompson, N.J., et al. (2005). Cotranscriptional set2 methylation of histone H3 lysine 36 recruits a repressive Rpd3 complex. Cell *123*, 593–605.

Kim, T., and Buratowski, S. (2009). Dimethylation of H3K4 by Set1 recruits the Set3 histone deacetylase complex to 5′ transcribed regions. Cell *137*, 259–272.

Kim, T.K., Ebright, R.H., and Reinberg, D. (2000). Mechanism of ATP-dependent promoter melting by transcription factor IIH. Science *288*, 1418–1422.

Kim, M., Vasiljeva, L., Rando, O.J., Zhelkovsky, A., Moore, C., and Buratowski, S. (2006). Distinct pathways for snoRNA and mRNA termination. Mol. Cell *24*, 723–734.

Kim, T., Xu, Z., Clauder-Münster, S., Steinmetz, L.M., and Buratowski, S. (2012). Set3 HDAC mediates effects of overlapping noncoding transcription on gene induction kinetics. Cell *150*, 1158–1169.

Kim, D., Pertea, G., Trapnell, C., Pimentel, H., Kelley, R., and Salzberg, S.L. (2013). TopHat2: accurate alignment of transcriptomes in the presence of insertions, deletions and gene fusions. Genome Biol. *14*, R36.

Kim, J.H., Lee, B.B., Oh, Y.M., Zhu, C., Steinmetz, L.M., Lee, Y., Kim, W.K., Lee, S.B., Buratowski, S., and Kim, T. (2016). Modulation of mRNA and lncRNA expression dynamics by the Set2-Rpd3S pathway. Nat. Commun. *7*, 13534.

Komarnitsky, P., Cho, E.J., and Buratowski, S. (2000). Different phosphorylated forms of RNA polymerase II and associated mRNA processing factors during transcription. Genes Dev. *14*, 2452–2460.

Krishnamurthy, S., He, X., Reyes-Reyes, M., Moore, C., and Hampsey, M. (2004). Ssu72 Is an RNA polymerase II CTD phosphatase. Mol. Cell *14*, 387–394.

Krogan, N.J., Kim, M., Tong, A., Golshani, A., Cagney, G., Canadien, V., Richards, D.P., Beattie, B.K., Emili, A., Boone, C., et al. (2003). Methylation of histone H3 by Set2 in Saccharomyces cerevisiae is linked to transcriptional elongation by RNA polymerase II. Mol. Cell. Biol. *23*, 4207–4218.

Langmead, B., and Salzberg, S.L. (2012). Fast gapped-read alignment with Bowtie 2. Nat. Methods *9*, 357–359.

Larochelle, M., Robert, M.A., Hébert, J.N., Liu, X., Matteau, D., Rodrigue, S., Tian, B., Jacques, P.É., and Bachand, F. (2018). Common mechanism of transcription termination at coding and noncoding RNA genes in fission yeast. Nat. Commun. *9*, 4364.

Lee, J.-H., You, J., Dobrota, E., and Skalnik, D.G. (2010). Identification and characterization of a novel human PP1 phosphatase complex. J. Biol. Chem. *285*, 24466–24476.

Lemay, J.-F., Marguerat, S., Larochelle, M., Liu, X., van Nues, R., Hunyadkürti, J., Hoque, M., Tian, B., Granneman, S., Bähler, J., and Bachand, F. (2016). The Nrd1-like protein Seb1 coordinates cotranscriptional 3′ end processing and polyadenylation site selection. Genes Dev. *30*, 1558–1572.

Lemieux, C., Marguerat, S., Lafontaine, J., Barbezier, N., Bähler, J., and Bachand, F. (2011). A Pre-mRNA degradation pathway that selectively targets intron-containing genes requires the nuclear poly(A)-binding protein. Mol. Cell *44*, 108–119.

Li, B., Howe, L., Anderson, S., Yates, J.R., III, and Workman, J.L. (2003). The Set2 histone methyltransferase functions through the phosphorylated carboxyl-terminal domain of RNA polymerase II. J. Biol. Chem. *278*, 8897–8903.

Li, B., Gogol, M., Carey, M., Lee, D., Seidel, C., and Workman, J.L. (2007). Combined action of PHD and chromo domains directs the Rpd3S HDAC to transcribed chromatin. Science *316*, 1050–1054.

Li, H., Handsaker, B., Wysoker, A., Fennell, T., Ruan, J., Homer, N., Marth, G., Abecasis, G., and Durbin, R.; 1000 Genome Project Data Processing Subgroup (2009). The Sequence Alignment/Map format and SAMtools. Bioinformatics *25*, 2078–2079.

Lock, A., Rutherford, K., Harris, M.A., Hayles, J., Oliver, S.G., Bähler, J., and Wood, V. (2019). PomBase 2018: user-driven reimplementation of the fission yeast database provides rapid and intuitive access to diverse, interconnected information. Nucleic Acids Res. *47*, D821–D827.

Love, M.I., Huber, W., and Anders, S. (2014). Moderated estimation of fold change and dispersion for RNA-seq data with DESeq2. Genome Biol. *15*, 550.

Lyons, D.E., McMahon, S., and Ott, M. (2020). A combinatorial view of old and new RNA polymerase II modifications. Transcription *11*, 66–82.

Mason, P.B., and Struhl, K. (2005). Distinction and relationship between elongation rate and processivity of RNA polymerase II in vivo. Mol. Cell *17*, 831–840.

Materne, P., Anandhakumar, J., Migeot, V., Soriano, I., Yague-Sanz, C., Hidalgo, E., Mignion, C., Quintales, L., Antequera, F., and Hermand, D. (2015). Promoter nucleosome dynamics regulated by signalling through the CTD code. eLife *4*, e09008.

Mayer, A., Lidschreiber, M., Siebert, M., Leike, K., Söding, J., and Cramer, P. (2010). Uniform transitions of the general RNA polymerase II transcription complex. Nat. Struct. Mol. Biol. *17*, 1272–1278.

Mayfield, J.E., Irani, S., Escobar, E.E., Zhang, Z., Burkholder, N.T., Robinson, M.R., Mehaffey, M.R., Sipe, S.N., Yang, W., Prescott, N.A., et al. (2019). Tyr1 phosphorylation promotes phosphorylation of Ser2 on the C-terminal domain of eukaryotic RNA polymerase II by P-TEFb. eLife *8*, 1–27.

Muñoz, M.J., de la Mata, M., and Kornblihtt, A.R. (2010). The carboxy terminal domain of RNA polymerase II and alternative splicing. Trends Biochem. Sci. *35*, 497–504.

Murray, S., Udupa, R., Yao, S., Hartzog, G., and Prelich, G. (2001). Phosphorylation of the RNA Polymerase II Carboxy-Terminal Domain by the Bur1 Cyclin-Dependent Kinase. Mol. Cell Biol. *21*, 4089–4096.

Neil, H., Malabat, C., d'Aubenton-Carafa, Y., Xu, Z., Steinmetz, L.M., and Jacquier, A. (2009). Widespread bidirectional promoters are the major source of cryptic transcripts in yeast. Nature *457*, 1038–1042.

Nemec, C.M., Singh, A.K., Ali, A., Tseng, S.C., Syal, K., Ringelberg, K.J., Ho, Y.H., Hintermair, C., Ahmad, M.F., Kar, R.K., et al. (2019). Noncanonical CTD kinases regulate RNA polymerase II in a gene-class-specific manner. Nat. Chem. Biol. *15*, 123–131.

Ng, H.H., Robert, F., Young, R.A., and Struhl, K. (2003). Targeted recruitment of Set1 histone methylase by elongating Pol II provides a localized mark and memory of recent transcriptional activity. Mol. Cell *11*, 709–719.

Ni, T., Tu, K., Wang, Z., Song, S., Wu, H., Xie, B., Scott, K.C., Grewal, S.I., Gao, Y., and Zhu, J. (2010). The prevalence and regulation of antisense transcripts in Schizosaccharomyces pombe. PLoS One *5*, e15271.

Nicolas, E., Yamada, T., Cam, H.P., Fitzgerald, P.C., Kobayashi, R., and Grewal, S.I.S. (2007). Distinct roles of HDAC complexes in promoter silencing, antisense suppression and DNA damage protection. Nat. Struct. Mol. Biol. *14*, 372–380.

Nielsen, S., Yuzenkova, Y., and Zenkin, N. (2013). Mechanism of eukaryotic RNA polymerase III transcription termination. Science *340*, 1577–1580.

Nojima, T., Tellier, M., Foxwell, J., Ribeiro de Almeida, C., Tan-Wong, S.M., Dhir, S., Dujardin, G., Dhir, A., Murphy, S., and Proudfoot, N.J. (2018). Deregulated Expression of Mammalian lncRNA through Loss of SPT6 Induces R-Loop Formation, Replication Stress, and Cellular Senescence. Mol. Cell *72*, 970–984.e7.

Pappas, D.L., Jr., and Hampsey, M. (2000). Functional interaction between Ssu72 and the Rpb2 subunit of RNA polymerase II in Saccharomyces cerevisiae. Mol. Cell. Biol. *20*, 8343–8351.

Parua, P.K., Booth, G.T., Sansó, M., Benjamin, B., Tanny, J.C., Lis, J.T., and Fisher, R.P. (2018). A Cdk9-PP1 switch regulates the elongation-termination transition of RNA polymerase II. Nature *558*, 460–464.

Pefanis, E., Wang, J., Rothschild, G., Lim, J., Chao, J., Rabadan, R., Economides, A.N., and Basu, U. (2014). Noncoding RNA transcription targets AID to divergently transcribed loci in B cells. Nature *514*, 389–393.

Pefanis, E., Wang, J., Rothschild, G., Lim, J., Kazadi, D., Sun, J., Federation, A., Chao, J., Elliott, O., Liu, Z.P., et al. (2015). RNA exosome-regulated long non-coding RNA transcription controls super-enhancer activity. Cell *161*, 774–789.

Pijnappel, W.W., Schaft, D., Roguev, A., Shevchenko, A., Tekotte, H., Wilm, M., Rigaut, G., Séraphin, B., Aasland, R., and Stewart, A.F. (2001). The S. cerevisiae SET3 complex includes two histone deacetylases, Hos2 and Hst1, and is a meiotic-specific repressor of the sporulation gene program. Genes Dev. *15*, 2991–3004.

Quinlan, A.R., and Hall, I.M. (2010). BEDTools: a flexible suite of utilities for comparing genomic features. Bioinformatics *26*, 841–842.

Ramírez, F., Ryan, D.P., Grüning, B., Bhardwaj, V., Kilpert, F., Richter, A.S., Heyne, S., Dündar, F., and Manke, T. (2016). deepTools2: a next generation web server for deep-sequencing data analysis. Nucleic Acids Res. *44*, W160–W165.

Rentas, S., Saberianfar, R., Grewal, C., Kanippayoor, R., Mishra, M., McCollum, D., and Karagiannis, J. (2012). The SET domain protein, Set3p, promotes the reliable execution of cytokinesis in Schizosaccharomyces pombe. PLoS One *7*, e31224.

Reppas, N.B., Wade, J.T., Church, G.M., and Struhl, K. (2006). The transition between transcriptional initiation and elongation in E. coli is highly variable and often rate limiting. Mol. Cell *24*, 747–757.

Rosonina, E., Yurko, N., Li, W., Hoque, M., Tian, B., and Manley, J.L. (2014). Threonine-4 of the budding yeast RNAP II CTD couples transcription with Htz1-mediated chromatin remodeling. Proc. Natl. Acad. Sci. USA *111*, 11924–11931.

Saldi, T., Cortazar, M.A., Sheridan, R.M., and Bentley, D.L. (2016). Coupling of RNA Polymerase II Transcription Elongation with Pre-mRNA Splicing. J. Mol. Biol. *428*, 2623–2635.

Sanchez, A.M., Garg, A., Shuman, S., and Schwer, B. (2019). Inositol pyrophosphates impact phosphate homeostasis via modulation of RNA 3′ processing and transcription termination. Nucleic Acids Res. *47*, 8452–8469.

Sanso, M., and Fisher, R.P. (2013). Modelling the CDK-dependent transcription cycle in fission yeast. Biochem. Soc. Trans. *41*, 1660–1665.

Sansó, M., Parua, P.K., Pinto, D., Svensson, J.P., Pagé, V., Bitton, D.A., MacKinnon, S., Garcia, P., Hidalgo, E., Bähler, J., et al. (2020). Cdk9 and H2Bub1 signal to Clr6-CII/Rpd3S to suppress aberrant antisense transcription. Nucleic Acids Res. *48*, 7154–7168.

Santos-Pereira, J.M., and Aguilera, A. (2015). R loops: new modulators of genome dynamics and function. Nat. Rev. Genet. *16*, 583–597.

Santos-Pereira, J.M., Herrero, A.B., García-Rubio, M.L., Marín, A., Moreno, S., and Aguilera, A. (2013). The Npl3 hnRNP prevents R-loop-mediated transcription-replication conflicts and genome instability. Genes Dev. *27*, 2445–2458.

Santos-Rosa, H., Schneider, R., Bannister, A.J., Sherriff, J., Bernstein, B.E., Emre, N.C., Schreiber, S.L., Mellor, J., and Kouzarides, T. (2002). Active genes are tri-methylated at K4 of histone H3. Nature *419*, 407–411.

Schaft, D., Roguev, A., Kotovic, K.M., Shevchenko, A., Sarov, M., Shevchenko, A., Neugebauer, K.M., and Stewart, A.F. (2003). The histone 3 lysine

36 methyltransferase, SET2, is involved in transcriptional elongation. Nucleic Acids Res. *31*, 2475–2482.

Schwer, B., Sanchez, A.M., and Shuman, S. (2012). Punctuation and syntax of the RNA polymerase II CTD code in fission yeast. Proc. Natl. Acad. Sci. USA *109*, 18024–18029.

Schwer, B., Bitton, D.A., Sanchez, A.M., Bähler, J., and Shuman, S. (2014). Individual letters of the RNA polymerase II CTD code govern distinct gene expression programs in fission yeast. Proc. Natl. Acad. Sci. USA *111*, 4185–4190.

Shah, S., Wittmann, S., Kilchert, C., and Vasiljeva, L. (2014). lncRNA recruits RNAi and the exosome to dynamically regulate pho1 expression in response to phosphate levels in fission yeast. Genes Dev. *28*, 231–244.

Sharma, V.M., Tomar, R.S., Dempsey, A.E., and Reese, J.C. (2007). Histone deacetylases RPD3 and HOS2 regulate the transcriptional activation of DNA damage-inducible genes. Mol. Cell. Biol. *27*, 3199–3210.

Shaw, R.J., and Reines, D. (2000). Saccharomyces cerevisiae transcription elongation mutants are defective in PUR5 induction in response to nucleotide depletion. Mol. Cell. Biol. *20*, 7427–7437.

Shetty, A., Kallgren, S.P., Demel, C., Maier, K.C., Spatt, D., Alver, B.H., Cramer, P., Park, P.J., and Winston, F. (2017). Spt5 Plays Vital Roles in the Control of Sense and Antisense Transcription Elongation. Mol. Cell *66*, 77–88.e5.

Shi, X., Kachirskaia, I., Walter, K.L., Kuo, J.H.A., Lake, A., Davrazou, F., Chan, S.M., Martin, D.G.E., Fingerman, I.M., Briggs, S.D., et al. (2007). Proteome-wide analysis in Saccharomyces cerevisiae identifies several PHD fingers as novel direct and selective binding modules of histone H3 methylated at either lysine 4 or lysine 36. J. Biol. Chem. *282*, 2450–2455.

Skourti-Stathaki, K., Kamieniarz-Gdula, K., and Proudfoot, N.J. (2014). R-loops induce repressive chromatin marks over mammalian gene terminators. Nature *516*, 436–439.

Soudet, J., Gill, J.K., and Stutz, F. (2018). Noncoding transcription influences the replication initiation program through chromatin regulation. Genome Res. *28*, 1882–1893.

Steinmetz, E.J., and Brow, D.A. (2003). Ssu72 protein mediates both poly(A)-coupled and poly(A)-independent termination of RNA polymerase II transcription. Mol. Cell. Biol. *23*, 6339–6349.

Tan-Wong, S.M., Zaugg, J.B., Camblong, J., Xu, Z., Zhang, D.W., Mischo, H.E., Ansari, A.Z., Luscombe, N.M., Steinmetz, L.M., and Proudfoot, N.J. (2012). Gene loops enhance transcriptional directionality. Science *338*, 671–675.

Taverna, S.D., Ilin, S., Rogers, R.S., Tanny, J.C., Lavender, H., Li, H., Baker, L., Boyle, J., Blair, L.P., Chait, B.T.T., et al. (2006). Yng1 PHD finger binding to H3 trimethylated at K4 promotes NuA3 HAT activity at K14 of H3 and transcription at a subset of targeted ORFs. Mol. Cell *24*, 785–796.

Tellier, M., Maudlin, I., and Murphy, S. (2020). Transcription and splicing: A two-way street. Wiley Interdiscip. Rev. RNA *11*, e1593.

Tietjen, J.R., Zhang, D.W., Rodríguez-Molina, J.B., White, B.E., Akhtar, M.S., Heidemann, M., Li, X., Chapman, R.D., Shokat, K., Keles, S., et al. (2010). Chemical-genomic dissection of the CTD code. Nat. Struct. Mol. Biol. *17*, 1154–1161.

Trapnell, C., Williams, B.A., Pertea, G., Mortazavi, A., Kwan, G., van Baren, M.J., Salzberg, S.L., Wold, B.J., and Pachter, L. (2010). Transcript assembly and quantification by RNA-Seq reveals unannotated transcripts and isoform switching during cell differentiation. Nat. Biotechnol. *28*, 511–515.

van Dijk, E.L., Chen, C.L., d'Aubenton-Carafa, Y., Gourvennec, S., Kwapisz, M., Roche, V., Bertrand, C., Silvain, M., Legoix-Né, P., Loeillet, S., et al. (2011). XUTs are a class of Xrn1-sensitive antisense regulatory non-coding RNA in yeast. Nature *475*, 114–117.

Vanoosthuyse, V., Legros, P., van der Sar, S.J.A., Yvert, G., Toda, K., Le Bihan, T., Watanabe, Y., Hardwick, K., and Bernard, P. (2014). CPF-associated phosphatase activity opposes condensin-mediated chromosome condensation. PLoS Genet. *10*, e1004415.

Vasiljeva, L., Kim, M., Mutschler, H., Buratowski, S., and Meinhart, A. (2008). The Nrd1-Nab3-Sen1 termination complex interacts with the Ser5-phosphorylated RNA polymerase II C-terminal domain. Nat. Struct. Mol. Biol. *15*, 795–804.

Venkatesh, S., Smolle, M., Li, H., Gogol, M.M., Saint, M., Kumar, S., Natarajan, K., and Workman, J.L. (2012). Set2 methylation of histone H3 lysine 36 suppresses histone exchange on transcribed genes. Nature *489*, 452–455.

Venkatesh, S., Li, H., Gogol, M.M., and Workman, J.L. (2016). Selective suppression of antisense transcription by Set2-mediated H3K36 methylation. Nat. Commun. *7*, 13610.

Vo, T.V., Dhakshnamoorthy, J., Larkin, M., Zofall, M., Thillainadesan, G., Balachandran, V., Holla, S., Wheeler, D., and Grewal, S.I.S. (2019). CPF Recruitment to Non-canonical Transcription Termination Sites Triggers Heterochromatin Assembly and Gene Silencing. Cell Rep. *28*, 267–281.e5.

Wang, A., Kurdistani, S.K., and Grunstein, M. (2002). Requirement of Hos2 histone deacetylase for gene activity in yeast. Science *298*, 1412–1414.

Watts, B.R., Wittmann, S., Wery, M., Gautier, C., Kus, K., Birot, A., Heo, D.H., Kilchert, C., Morillon, A., and Vasiljeva, L. (2018). Histone deacetylation promotes transcriptional silencing at facultative heterochromatin. Nucleic Acids Res. *46*, 5426–5440.

Wery, M., Gautier, C., Descrimes, M., Yoda, M., Vennin-Rendos, H., Migeot, V., Gautheret, D., Hermand, D., and Morillon, A. (2018). Native elongating transcript sequencing reveals global anti-correlation between sense and antisense nascent transcription in fission yeast. RNA *24*, 196–208.

Wirén, M., Silverstein, R.A., Sinha, I., Walfridsson, J., Lee, H.M., Laurenson, P., Pillus, L., Robyr, D., Grunstein, M., and Ekwall, K. (2005). Genomewide analysis of nucleosome density histone acetylation and HDAC function in fission yeast. EMBO J. *24*, 2906–2918.

Wittmann, S., Renner, M., Watts, B.R., Adams, O., Huseyin, M., Baejen, C., El Omari, K., Kilchert, C., Heo, D.-H., Kecman, T., et al. (2017). The conserved protein Seb1 drives transcription termination by binding RNA polymerase II and nascent RNA. Nat. Commun. *8*, 14861.

Xiang, K., Nagaike, T., Xiang, S., Kilic, T., Beh, M.M., Manley, J.L., and Tong, L. (2010). Crystal structure of the human symplekin-Ssu72-CTD phosphopeptide complex. Nature *467*, 729–733.

Xiang, K., Manley, J.L., and Tong, L. (2012). An unexpected binding mode for a Pol II CTD peptide phosphorylated at Ser7 in the active site of the CTD phosphatase Ssu72. Genes Dev. *26*, 2265–2270.

Xu, Z., Wei, W., Gagneur, J., Perocchi, F., Clauder-Münster, S., Camblong, J., Guffanti, E., Stutz, F., Huber, W., and Steinmetz, L.M. (2009). Bidirectional promoters generate pervasive transcription in yeast. Nature *457*, 1033–1037.

Yamanaka, S., Mehta, S., Reyes-Turcu, F.E., Zhuang, F., Fuchs, R.T., Rong, Y., Robb, G.B., and Grewal, S.I.S. (2013). RNAi triggered by specialized machinery silences developmental genes and retrotransposons. Nature *493*, 557–560.

Yurko, N.M., and Manley, J.L. (2018). The RNA polymerase II CTD "orphan" residues: Emerging insights into the functions of Tyr-1, Thr-4, and Ser-7. Transcription *9*, 30–40.

Zaborowska, J., Egloff, S., and Murphy, S. (2016). The pol II CTD: new twists in the tail. Nat. Struct. Mol. Biol. *23*, 771–777.

Zhang, D.W., Mosley, A.L., Ramisetty, S.R., Rodríguez-Molina, J.B., Washburn, M.P., and Ansari, A.Z. (2012). Ssu72 phosphatase-dependent erasure of phospho-Ser7 marks on the RNA polymerase II C-terminal domain is essential for viability and transcription termination. J. Biol. Chem. *287*, 8541–8551.

CellPress
OPEN ACCESS

STAR★METHODS

KEY RESOURCES TABLE

REAGENT or RESOURCE	SOURCE	IDENTIFIER
Antibodies		
anti-Pol II (8WG16) (mouse)	Millipore	Cat#05-952; RRID: AB_492629
anti-Pol II CTD phospho Ser2, Clone 3E10 (rat)	Millipore	Cat#04-1571-I; RRID: AB_11212363
anti-Pol II CTD phospho Ser5, Clone 3E8 (rat)	Millipore	Cat#04-1572-I; RRID: AB_10615822
anti-Pol II CTD phospho Tyr1, Clone 3D12 (rat)	Active Motif	Cat#61383; RRID: AB_2793613
anti-Pol II CTD phospho Ser7, Clone 4E12 (rat)	Active Motif	Cat#61087; RRID: AB_2687452
Anti-Histone H4 Antibody, pan, clone 62-141-13 (rabbit)	Upstate/Millipore	Cat#05-858; RRID: AB_390138
Anti-acetyl-Histone H4 Antibody (rabbit)	Upstate/Millipore	Cat#06-598; RRID: AB_2295074
Anti-HA tag antibody - ChIP Grade (rabbit)	Abcam	Cat#ab9110; RRID: AB_307019
Chemicals, peptides, and recombinant proteins		
6-azauracil	Sigma-Aldrich	CAS: 461-89-2
Methyl methanesulfonate (MMS)	Alpha Aesar	H55120; CAS: 66-27-3
Hydroxyurea (HU)	Sigma-Aldrich	H8627; CAS: 127-07-1
Anti-Flag M2 affinity gel	Sigma-Aldrich	Cat#A2220; RRID: AB_10063035
Protein G Dynabeads	ThermoFisher	Cat#10004D
Streptavidin Sepharose™ High Performance affinity resin	Cytvia	Cat#17511301
Critical commercial assays		
NEBNext Ultra DNA library Kit for Illumina	NEB	Cat#E73705
NEBNext Fast DNA Library Prep Set for Ion Torrent	NEB	Cat#E62705
Deposited data		
Raw and analyzed data (RNA- and ChIP-Seq)	This manuscript	GEO: GSE144603 and GSE171307
Experimental models: Organisms/strains		
Schizosaccharomyces pombe strains generated by this study are YP144 background (h+, leu1-32, ura4Δ18, ade6-M216, his3Δ::1).	This manuscript (See Table S6)	N/A
Saccharomyces cerevisiae strain used by this study (as a spike in for RNA-seq and ChIP-seq experiments) is BY4741 (YF336) (MATa, ura3Δ0, leu2Δ0, his3Δ1, met15Δ0).	Saccharomyces Genome Deletion Project (See Table S6)	N/A
Oligonucleotides		
Multiple oligonucleotide sequences	This manuscript (See Table S7)	N/A
Software and algorithms		
Bowtie2	Langmead and Salzberg, 2012	http://bowtie-bio.sourceforge.net/bowtie2/index.shtml
Samtools	Li et al., 2009	http://samtools.sourceforge.net/
deepTools	Ramírez et al., 2016	https://deeptools.readthedocs.io/en/develop/
Python3.8	Python Software Foundation	https://www.python.org/

(Continued on next page)

Continued

REAGENT or RESOURCE	SOURCE	IDENTIFIER
R	The R Foundation	https://www.r-project.org/
DESeq2	Love et al., 2014	https://bioconductor.org/packages/release/bioc/html/DESeq2.html
Trimmomatic	Bolger et al., 2014	http://www.usadellab.org/cms/?page=trimmomatic
fastp	Chen et al., 2018	https://github.com/OpenGene/fastp
TopHat2	Kim et al., 2013	https://ccb.jhu.edu/software/tophat/index.shtml
STAR	Dobin et al., 2013	https://github.com/alexdobin/STAR
Cufflinks	Trapnell et al., 2010	http://cole-trapnell-lab.github.io/cufflinks/
bedtools	Quinlan and Hall, 2010	https://bedtools.readthedocs.io/en/latest/

RESOURCE AVAILABILITY

Lead contact

Further information and requests for resources and reagents should be directed to and will be fulfilled by the Lead Contact, Lidia Vasiljeva (lidia.vasilieva@bioch.ox.ac.uk).

Materials availability

All unique reagents generated in this study are available from the Lead Contact upon request.

Data and code availability

- All raw sequencing data used in this study were deposited at GEO under accession numbers GEO: GSE144603 and GEO: GSE171307. They are publicly available as of the date of publication. Accession number is also listed in the Key resources table.
- This study did not generate any code.
- Any additional information required to reanalyse the data reported in this paper is available from the Lead Contact upon request.

EXPERIMENTAL MODEL AND SUBJECT DETAILS

Yeast strains

Chromosomally tagged *Schizosaccharomyces pombe* strains and mutants were constructed by a PCR-based strategy, by genetic crosses and standard techniques (Bähler et al., 1998). Strains and all genetic manipulations were verified by polymerase chain reaction (PCR), sequencing and phenotype. All yeast strains used in this work except those derived from the strains obtained from other labs are isogenic to YP144 background and are listed in the Key resources table and Table S6.

METHOD DETAILS

Yeast techniques

Yeast cultures were inoculated from overnight cultures, grown using standard growth conditions and media (Forsburg, 2003). For ChIP-Seq, RNA-Seq, Northern blot yeast strains were grown in YES media (0.5% yeast extract, 3% glucose, 0.0225% supplements (Histidine, Adenine, Uracil, Leucine, Lysine) at 30°C to OD_{600} of 0.6~0.7. For drug sensitivity assays, cells from overnight cultures were counted and diluted before being spotted on plates containing the indicated concentrations of drugs. To test the sensitivity of the strains to 6-AU, WT and *ssu72Δ* were grown on minimal media lacking uracil with DMSO or 6-AU (500 μg/ml) as indicated. The sensitivity of cells to DNA damaging agents was tested on YES media with or without 0.01% of MMS (Alpha Aesar, H55120) or 5, 10 mM HU (Sigma-Aldrich, H8627). Serial dilutions of the cells were plated onto indicated media. The cells were grown for 4 days at indicated temperatures.

Chromatin immunoprecipitation (ChIP-Seq and ChIP-qPCR)

All ChIP-Seq experiments were carried out in duplicates except Ser7-P ChIP-Seq. Chromatin was prepared as previously described (Kecman et al., 2018b). Briefly, exponentially growing cells (200 ml) were crosslinked with 1% formaldehyde solution for 20 min at

room temperature. For spike-in control, *S. cerevisiae* cells were added to *S. pombe* cells at a 1:10 ratio before cross-linking. Thirty milliliters of a solution of 3M glycine, 20 mM Tris-HCl, pH 7.5 was used to quench the reaction. Cells were pelleted and washed once with cold TBS and once with FA lysis buffer (50 mM HEPES-KOH pH 7.5, 150 mM NaCl, 1 mM EDTA, 1% Triton X-100, 0.1% Na Deoxycholate) with 0.1% SDS. To prepare chromatin, cells were resuspended in FA lysis buffer with 0.5% SDS and vortexed for 30 cycles of 1 min vortexing and 1 min on ice. The lysate was ultracentrifuged (150,000 g, 20 min) and the pellet crushed in lysis buffer. Samples were sheared for 80 min with a sonication cycle of 15 s ON/45 s OFF with a Biorupter sonicator, and ultracentrifuged (150,000 g, 20 min) to yield sheared chromatin (300-500 bp DNA fragments) in the supernatant. At this point the concentration of NaCl was adjusted to 275 mM.

Immunoprecipitation was carried out using antibodies against Rpb1 (8WG16, Millipore), Ser2-P CTD (3E10, Millipore), Ser5-P CTD (3E8, Millipore), Tyr1-P CTD (3D12, Active Motif) and Ser7-P CTD (4E12, Active Motif) preincubated with protein G Dynabeads (ThermoFisher) and antibodies against H4 (05-858, Upstate/Millipore), H4KAc (06-598, Upstate/Millipore) and Flag (A2220, Millipore) and HA (ab9110, Abcam) epitope tags. After washing and eluting bound material from the beads, protein was removed by incubation with 0.2 mg pronase for 1 h at 42 °C, followed by overnight incubation at 65 °C. RNA was degraded by incubating samples with 0.02 mg RNase A (Roche) for 1 h at 37 °C. DNA was then purified using ChIP DNA Clean & Concentrator kit (Zymo Research, USA) according to the manufacturer's instructions.

Libraries were prepared using NEBNext Fast DNA Library Prep Set for Ion Torrent kit for Rpb1, Ser2-P and Tyr1-P or NEBNext Ultra DNA Library Prep Kit for Illumina for Ser5-P and Ser7-P. The libraries were sequenced on the Ion Torrent Proton or the Illumina Next-Seq 500. For ChIP-qPCR, PCR was carried out using primer pairs from Table S7 as shown in Figures 6A, 6C, and S3E. The number of replicates is indicated in the figure legends.

ChIP-Seq data analysis

Sequenced reads were trimmed by Trimmomatic (Bolger et al., 2014), aligned to a concatenated genome (*S. pombe* + *S. cerevisiae*) using Bowtie2 (Langmead and Salzberg, 2012), and reads were separated by species-specific chromosome names. After the removal of reads mapped more than once, uniquely mapped reads were obtained by SAMtools (Li et al., 2009). To assess changes in protein occupancy between strains, a spike-in normalization was used where *S. pombe* reads were adjusted using the number of *S. cerevisiae* reads in each IP sample. Genomic DNA sequencing of the input mixture of fission and budding yeast was also used to correct for any variation in cell mixture ratios. Bigwig and bedgraph files were generated by deepTools (Ramírez et al., 2016). Genome annotation was used from the study by Eser et al. (2016). For heatmaps shown in Figures 1 and 2, custom scripts were used. For metagene and traveling ratio analysis quantifying the ratio between the promoter (200 bp downstream of TSS) and the terminator proximal signal (200 bp upstream of poly(A) site - PAS) of RNApII, the number of reads within specified regions were calculated by deepTools (Ramírez et al., 2016) and plotted using custom scripts.

RNA sequencing (RNA-Seq)

For spike-in normalization, the *S. cerevisiae* cells were added to *S. pombe* at 1:10 ratio prior to RNA isolation except series of experiments for *rpb1*-CTD-S2A where purified RNA was used instead. Total RNA was extracted using a standard hot phenol method and treated with RNase-free DNase RQ1 (Promega) to remove any DNA contamination. Libraries for RNA-Seq experiments were prepared and sequenced by the High-throughput Genomics Group at the Wellcome Trust Centre for Human Genetics using the Illumina HiSeq 2500 platform for WT and *ssu72*Δ strains. For the second batch of experiments, RNA isolated from WT, *ssu72*Δ, *hos2*Δ and *ssu72*Δ*hos2*Δ strains was depleted from rRNA using Ribo-Zero rRNA removal kit (Illumina) and then libraries were prepared using NEBNext Ultra Directional RNA Library Prep Kit for Illumina (NEB) and sequenced on the Illumina NextSeq 500. These experiments showed lower coverage, which affected the number of antisense transcripts detected (212 antisense transcripts were found to increase more than 1.5 times in *ssu72*Δ compared to WT (in contrast to the first batch of experiments /WT and *ssu72*Δ only/ with 519 antisense above this threshold). Nevertheless, high concordance is observed in assigned upregulated antisense in *ssu72*Δ comparing first and second RNA-Seq batches.

RNA-Seq data analysis

Sequenced reads were trimmed using Trimmomatic or fastp (Bolger et al., 2014; Chen et al., 2018), aligned to a concatenated genome using TopHat2 or STAR (Dobin et al., 2013; Kim et al., 2013). Reads were separated by species-specific chromosome names. Uniquely mapped reads were extracted using SAMtools (Li et al., 2009). Fission yeast genome annotation from (Eser et al., 2016) was used in downstream analyses except for analyses to identify potential 3′ read-through cases where annotation was extended with TUs from the Pombase database (Lock et al., 2019). Differential gene expression analysis was carried out using DESeq2, an R package (Love et al., 2014). Gene ontology analysis was performed using a web-based tool AnGeLi (Bitton et al., 2015). Metagenes were prepared with deepTools (Ramírez et al., 2016). For scaled metagenes, a matrix generated by deepTools was used to transform the signal of each TU to range 0-1 with a custom python script and this matrix served for the final metagene plot. Changes in the phosphorylation patterns were also evaluated with deepTools and plotted with custom python scripts. Random selection of genes was repeated several times, results were comparable, representative plots were selected. Statistical analysis was performed using python libraries.

Identification of novel transcripts

All transcripts were assembled from uniquely mapped reads obtained from two replicates of WT and *ssu72Δ* using the Cufflinks program (Trapnell et al., 2010; Venkatesh et al., 2016). Transcripts on each strand were merged if reads were not separated by more than 5 nt. To keep only transcripts expressed in both biological replicates, BEDTools were used using "intersect -s" option (Quinlan and Hall, 2010). All transcripts obtained from WT and *ssu72Δ* were combined by conventional Linux command and the resultant transcripts were sorted and merged by BEDTools. The novel transcripts were chosen to have no more than 10% overlap with any annotated TU reciprocally by BEDTools using "intersect -wa -s -v -f 0.1 -r" options. Transcripts that were longer than 5 kb or shorter than 50 bp were filtered out. The transcripts suppressed by Ssu72 were collected based on abundance (> 1FPKM) and fold change (> $\log_2(1.5)$) compared to WT using a custom script and DESeq2 (Love et al., 2014). Surprisingly, the length of the predicted antisense transcripts tends to be comparable to the length of the overlapping annotated TU. The majority of the predicted novel TSS (245 out of predicted 277 transcripts, 88.4%) were located within ± 300 nt from annotated PAS whereas only 13% (36 out of 277) of predicted TSS' were within the window of annotated TSS on the opposite strand. Only minimal overlap was observed between novel transcripts and XUTs (van Dijk et al., 2011; Wery et al., 2018).

Identification of genes with potential 3′ read-through transcription

Pooled annotation from Eser et al. (2016) and Lock et al. (2019) was filtered to exclude TUs that have another gene on the same strand in the 250bp window upstream TSS or downstream PAS. For each TU RNA-Seq read density was calculated in a 250 bp window downstream PAS using deepTools. The signal for biological replicates was averaged and the standard deviation was calculated. The ratio between *ssu72Δ* and WT in the read density downstream PAS was corrected for the difference in the density of the reads upstream PAS (also calculated in 250bp window, to account for gene expression changes). If the corrected signal downstream PAS was higher than two-fold between *ssu72Δ* and WT, the normalized standard deviation was lower than 0.6, WT cells had at least 10 reads in the region upstream PAS and *ssu72Δ* had at least 5 reads downstream PAS, the gene was selected to have increased in the read-through and resultant 507 cases have been identified. This group includes protein-coding (n = 441), multicistronic (n = 37) and non-coding (n = 29) genes. The actual number of genes dependent on Ssu72 for transcription termination might be higher since we excluded genes with other TUs nearby on the same strand and selected genes that show > 2-fold increase in the 3′ reads. Indeed, Ssu72 was proposed to contribute to transcription termination of ncRNA *prt* (Vo et al., 2019) produced from the promoter located upstream of the *pho1* gene (Shah et al., 2014; Yamanaka et al., 2013), which is excluded from our analyses due to above-mentioned requirements. Reducing stringency of selection (> 1.5-fold increase in 3′ reads) leads to the identification of the higher number of TUs with the read-through transcription (n = 896).

Northern blot

Total RNA was extracted by a hot phenol method. 10 μg of RNA was loaded per lane and resolved on 1.2% agarose gel containing 6.7% formaldehyde in MOPS buffer. After capillary transfer in 10 × SSC onto Hybond N+ membranes (GE Healthcare), RNA was UV-crosslinked and stained with methylene blue. Gene-specific probes were generated by PCR using oligos listed in Table S7 and labeled by random priming in the presence of [α-^{32}P] ATP using the Prime-It II Random Primer Labeling Kit (Agilent). The membrane was hybridized at 42 °C overnight. After repeated washes in 2 × SSC, 0.1% SDS, blots were exposed on Amersham Hyperfilm MP (GE Healthcare) or quantified with a FLA-7000 phosphoimager (Fujifilm). Methylene blue was used to visualize rRNA.

Hos2 purification

3x Flag-tagged Hos2 was purified from 20 L of yeast culture grown in YES to OD_{600} of 1.8 ~2. The cells were disrupted by a freezer mill (SPEX SamplePrep). The cell lysate was clarified by centrifugation at 30,000*g* for 45 mins and incubated with 1 mL of M2 beads (Sigma) in binding buffer (50 mM Tris-HCl, pH 7.5, 150 mM NaCl, 0.5 mM DTT, 0.5 mM $MgCl_2$, 0.5 mM $Zn(OAc)_2$, 0.5 mM $Mg(OAc)_2$, 0.5% Triton X-100, 10% glycerol) containing a proteinase inhibitor cocktail for 2 hr at 4°C. Beads were washed 3 times with lysis buffer and 3 times with washing buffer (50 mM Tris-HCl (pH 7.5), 300 mM NaCl, 0.5 mM DTT, 0.5 mM $MgCl_2$, 0.5 mM $Zn(OAc)_2$, 0.5 mM $Mg(OAc)_2$, 10% glycerol), then Hos2 complex were eluted with 2 mL of 5 mg/ml Flag peptide (Sigma) in elution buffer (25 mM Tris-HCl, pH 7.5, 150 mM NaCl, 3 mM DTT, 0.5 mM $MgCl_2$, 0.5 mM $Mg(OAc)_2$, 10% glycerol). Samples were concentrated using Amicon Ultra Centrifugal Filters (3kDa) and subjected to gel filtration using Superose 6 Increase 10/300 GL equilibrated with 20 mM HEPES, pH 7.6, 150 mM NaCl, 1 mM β-mercaptoethanol, 0.5 mM $MgCl_2$, 0.5 mM $Mg(OAc)_2$. Hos2 complex was resolved on NuPAGE 4%–12% Bis-Tris gel (Thermo Fisher scientific), visualized by silver staining and identified by mass spectrometry (Oxford Advanced Proteomics Facility).

In vitro transcription

To purify RNApII, 3xFlag or 5xFlag tag was introduced on Rpb9 or Rpb3 subunits in the WT or Ssu72 deficient cells, respectively. Mutant *ssu72Δ* and WT cells were grown to 0.9 OD_{600}. Pellets from 20 L per strain were mechanically disrupted in liquid nitrogen using a freezer mill (SPEX SamplePrep). Powder was mixed with 100 mL of lysis buffer (50 mM Tris-HCl (pH 7.6), 150 mM NaCl, 10% glycerol, 0.5% Triton X-100, 0.5 mM DTT, 0.5 mM $MgCl_2$, 0.5 mM $Mg(OAc)_2$) supplemented with a proteinase inhibitor cocktail and 0.4 mM final sodium orthovanadate. The lysate was centrifuged for 20 mins at 40000 g and incubated with 4 mL of ANTI-FLAG® M2 Affinity Gel slurry (Sigma) (equilibrated with lysis buffer) for 1.5 hr. Beads were washed four times with 15 mL of wash buffer

(50 mM Tris-HCl (pH 7.5), 1 M NaCl, 1 M urea, 10% glycerol, 0.5% Triton X-100, 0.5 mM DTT, 0.5 mM $MgCl_2$, 0.5 mM $Mg(OAc)_2$) and 4 times with low salt buffer buffer (50 mM Tris-HCl (pH 7.6), 150 mM NaCl, 10% glycerol, 0.05% Triton X-100, 0.5 mM DTT, 0.5 mM $MgCl_2$, 0.5 mM $Mg(OAc)_2$). RNApII was eluted twice with 2.5 mL of 5 mg/ml Flag-peptide (Sigma) in 50 mM Tris-HCl (pH 7.5), 150 mM NaCl, 10% glycerol, 0.5 mM DTT, 0.5 mM $MgCl_2$, 0.5 mM $Mg(OAc)_2$. Eluted RNApII was mixed with 45 mL of QA buffer (50 mM Tris-HCl (pH 7.5), 5 mM NaCl, 10% glycerol, 0.5 mM $MgCl_2$, 0.5 $Mg(OAc)_2$, 1 mM β-mercaptoethanol) to decrease salt concentration. Protein was further purified on the ion exchange chromatography column (2x 1ml HiTrap Q HP, GE Healthcare) equilibrated with QA buffer. The column was washed with several column volumes of 8% buffer QB (same as QA, except 2000 mM NaCl) until a stable baseline was achieved. RNApII was eluted with a gradient of QB buffer (up to 60%). Fractions containing RNApII were mixed and diluted with 75% glycerol to reach 50% and stored at $-20^{\circ}C$ until use.

Elongation complexes were assembled in transcription buffer, TB (20 mM Tris-HCl (pH 7.9), 0.2 mM EDTA, 40 mM KCl) using 5 nM RNApII, 50 nM 13 nt long RNA (RNA13), 50 nM template (T-REB37) and 500 nM non-template (NT-REB37-Biotin) DNA strands (IDT, details of sequences in Table S7), and complexes immobilised on Streptavidin Sepharose^{TM} High Performance beads (Cytiva) via biotin on non-template strand as previously described (Nielsen et al., 2013). RNA was labeled during walking with [α-^{32}P] CTP and [α-^{32}P] ATP (Hartmann Analytic) to produce RNA17. Beads were washed with TB containing 0.5 M KCl and then with TB. Transcription was started by the addition of NTPs to concentrations indicated in the figure and 10 mM $MgCl_2$ and carried out at $30^{\circ}C$ for times indicated in the figure. Reactions were stopped with formamide-containing buffer, and products resolved by denaturing PAGE and visualized using Phosphorimager (Typhoon Trio; GE Healthcare Life Sciences).

QUANTIFICATION AND STATISTICAL ANALYSIS

All ChIP-seq, RNA-seq were performed in at least two biological replicas. Statistical analysis was done using Mann–Whitney U test for independent, and Wilcoxon signed-rank test for matching gene sets and is indicated in the corresponding figure legends. Correction for multiple testing was performed using the Bonferroni method if required. Data were presented as medians or means. Error bars represent the standard error of the mean (SEM) and this information is provided in the figure legends.

Cell Reports, Volume *36*

Supplemental information

Transcription and chromatin-based surveillance mechanism controls suppression of cryptic antisense transcription

Dong-Hyuk Heo, Krzysztof Kuś, Pawel Grzechnik, Sue Mei Tan-Wong, Adrien Birot, Tea Kecman, Soren Nielsen, Nikolay Zenkin, and Lidia Vasiljeva

Figure S1

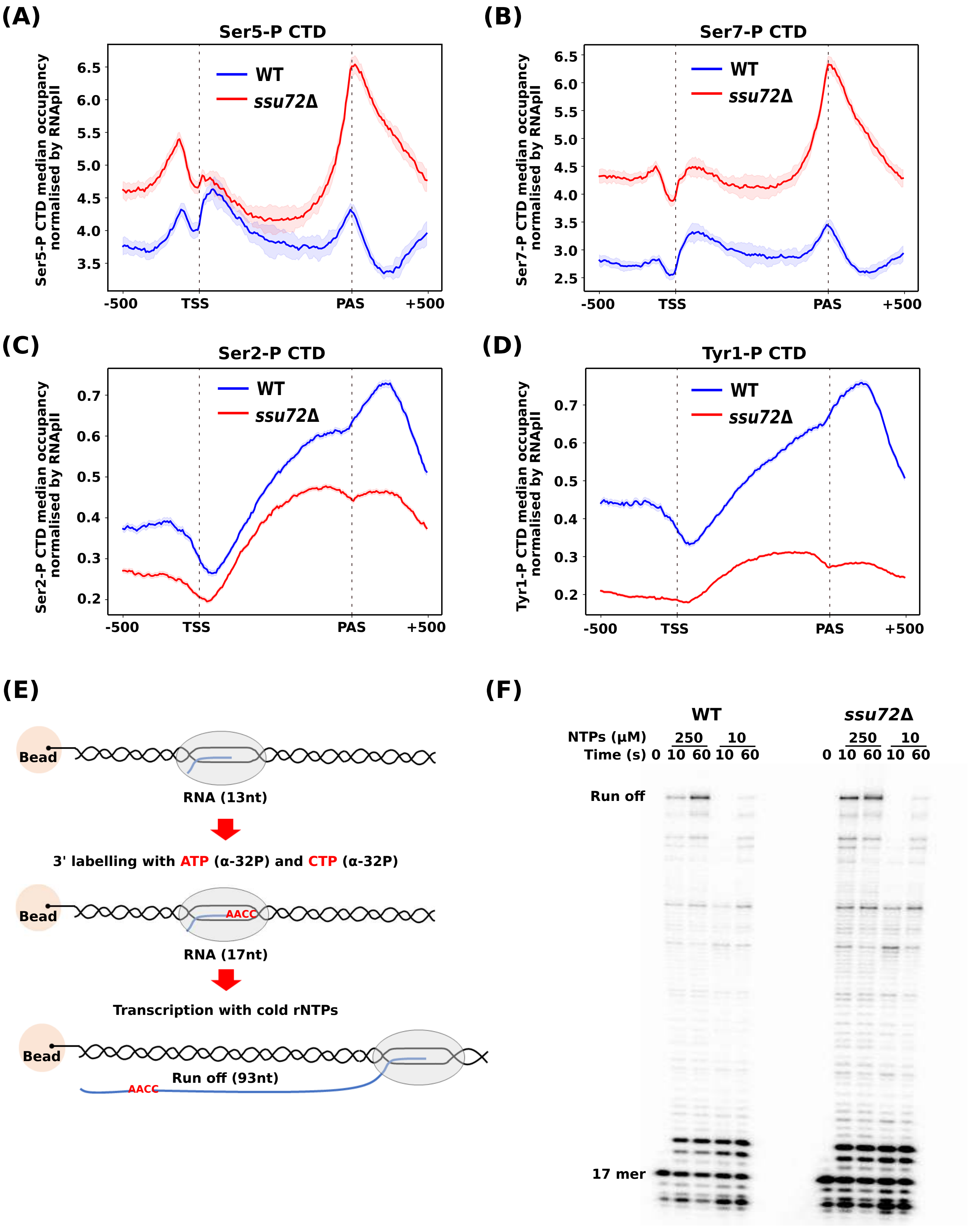

(A)
Ser5-P CTD
Ser5-P CTD median occupancy normalised by RNApII
WT
ssu72Δ
-500
TSS
PAS
+500
(B)
Ser7-P CTD
Ser7-P CTD median occupancy normalised by RNApII
WT
ssu72Δ
(C)
Ser2-P CTD
Ser2-P CTD median occupancy normalised by RNApII
WT
ssu72Δ
(D)
Tyr1-P CTD
Tyr1-P CTD median occupancy normalised by RNApII
WT
ssu72Δ
(E)
Bead
RNA (13nt)
3' labelling with ATP (α-32P) and CTP (α-32P)
Bead
AACC
RNA (17nt)
Transcription with cold rNTPs
Bead
Run off (93nt)
AACC
(F)
WT
ssu72Δ
NTPs (μM) 250 10 250 10
Time (s) 0 10 60 10 60 0 10 60 10 60
Run off
17 mer

Figure S1. Ssu72 modifies RNApII CTD phosphorylation pattern *in vivo* but changes do not alter enzymatic properties of purified RNApII *in vitro*. Related to Figures 1 and 2.

(A to D) Metagene profiles of RNApII CTD phosphorylation (Ser5-P in (A), Ser7-P in (B), Ser2-P in (C), and Tyr1-P in (D)) in WT (blue) and *ssu72*Δ (red) strains generated using 10-bp bins within ±500 bp around TSS and PAS. The signal was normalised by RNApII occupancy (8WG16 antibody). The shaded regions represent 95% confidence intervals.

(E) A schematic diagram describing *in vitro* transcription assay.

(F) RNAs transcribed by RNApII purified from WT and *ssu72*Δ strains were resolved in 10% denaturating UREA-TBE acrylamide gel and detected by phosphorimaging.

Figure S2

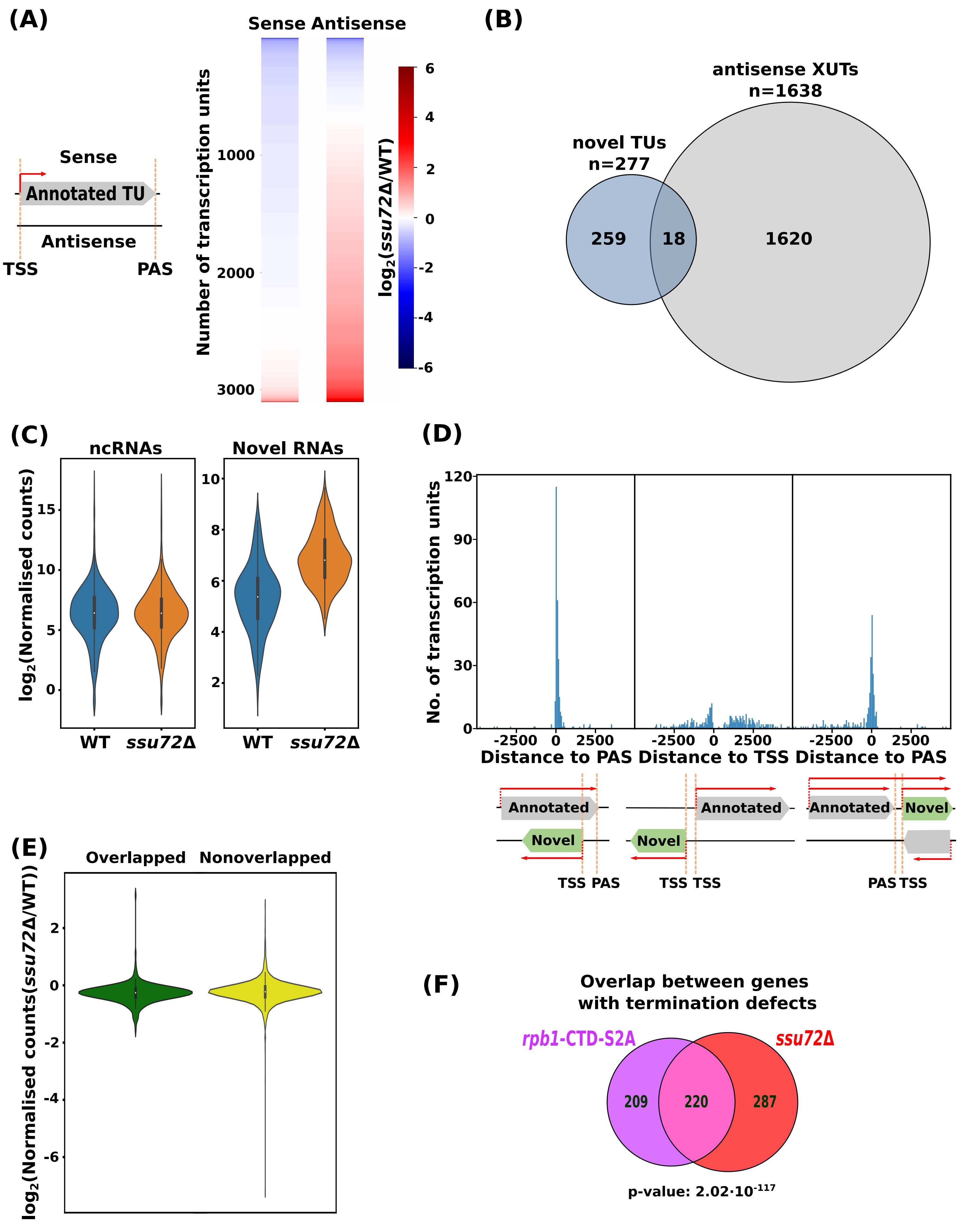

(A)
Sense Antisense
Sense
Annotated TU
Antisense
TSS
PAS
Number of transcription units
1000
2000
3000
log2(ssu72Δ/WT)
6
4
2
0
-2
-4
-6
(B)
antisense XUTs
n=1638
novel TUs
n=277
259
18
1620
(C)
ncRNAs
Novel RNAs
log2(Normalised counts)
WT
ssu72Δ
(D)
No. of transcription units
120
90
60
30
0
-2500 0 2500
Distance to PAS
Distance to TSS
Distance to PAS
Annotated
Novel
TSS PAS
TSS TSS
PAS TSS
(E)
Overlapped
Nonoverlapped
log2(Normalised counts(ssu72Δ/WT))
(F)
Overlap between genes
with termination defects
rpb1-CTD-S2A
ssu72Δ
209
220
287
p-value: 2.02·10-117

Figure S2. Deletion of Ssu72 induces cryptic antisense transcripts and readthrough. Related to Figures 4 and 5.
(A) A schematic diagram showing sense strand (coding strand) and antisense strand (template strand) of annotated non-overlapping transcripts units (left panel). Transcripts derived from the coding strand (sense) and the opposite strand (antisense) were sorted based on the fold change (log_2) in the levels in the Ssu72 mutant compared to WT – first batch of experiments (compare Figure 5D, Method section).
(B) Venn diagram showing the overlap between 277 antisense transcripts dependent on Ssu72 and Xrn1–regulated antisense transcripts from (Wery et al., 2018).
(C) Violin plots show changes in the levels of the annotated ncRNAs (left), and novel RNAs (right) in WT and *ssu72*Δ (the spike-in normalised log_2 value – first batch of experiments).
(D) The histogram depicts the distribution of distances from the predicted TSS of novel Ssu72-dependent transcripts (n=277) to the closest PAS and TSS of the annotated TU.
The diagram shows three scenarios: novel transcript starts from the opposite strand within the region close to PAS of the annotated gene (left), novel TSS is closest to the annotated TSS (middle) in the opposite strand, and to annotated PAS (right) on the same strand.
(E) The violin plot analyses assessing changes in the levels of the annotated transcripts overlapping and non-overlapping with antisense transcripts in *ssu72*Δ (log_2 value).
(F) Venn diagram indicating overlap between genes with termination defects in *ssu72*Δ and *rpb1*-CTD-S2A.

Figure S3

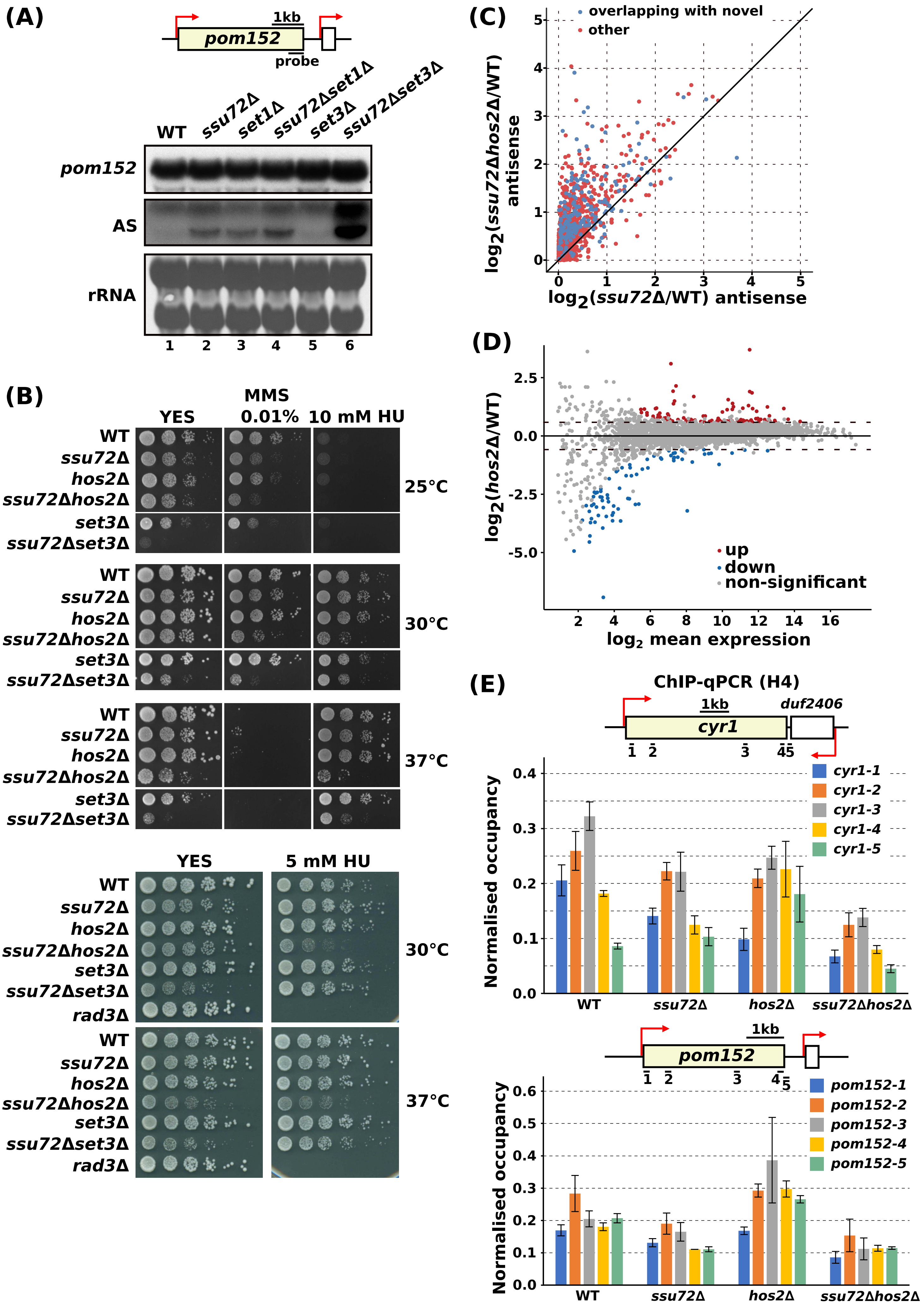
(A)
pom152
1kb
probe
WT
ssu72Δ
set1Δ
ssu72Δset1Δ
set3Δ
ssu72Δset3Δ
AS
rRNA
1 2 3 4 5 6
(B)
YES
MMS 0.01%
10 mM HU
WT
ssu72Δ
hos2Δ
ssu72Δhos2Δ
set3Δ
ssu72Δset3Δ
25°C
30°C
37°C
5 mM HU
rad3Δ
(C)
overlapping with novel
other
log2(ssu72Δhos2Δ/WT) antisense
log2(ssu72Δ/WT) antisense
(D)
log2(hos2Δ/WT)
log2 mean expression
up
down
non-significant
(E)
ChIP-qPCR (H4)
1kb
duf2406
cyr1
cyr1-1
cyr1-2
cyr1-3
cyr1-4
cyr1-5
Normalised occupancy
WT
ssu72Δ
hos2Δ
ssu72Δhos2Δ
pom152
pom152-1
pom152-2
pom152-3
pom152-4
pom152-5

Figure S3. Hos2 enhances Ssu72-regulated molecular phenotypes. Related to Figures 5 and 6.
(A) Levels of *pom152* mRNA and *pom152* antisense (AS) RNA were analysed by Northern blot in the indicated strains. Methylene blue staining was used to visualize rRNA as a loading control.
(B) Analyses of cellular growth in the presence of genotoxic agents (MMS and HU). The growth of *ssu72*Δ*hos2*Δ is compromised in the presence of HU. Indicated strains (WT, *ssu72*Δ, *hos2*Δ, *set3*Δ, *ssu72*Δ*hos2*Δ and *ssu72*Δ*set3*Δ, *rad3*Δ) were grown at 25°C, 30°C or 37°C temperatures in YES media without or with indicated concentration of drugs.
(C) Hos2 limits Ssu72-dependent antisense transcripts globally. Scatter plot showing the log_2-fold change in the levels of antisense in *ssu72*Δ (x-axis) and *ssu72*Δ *hos2*Δ (y-axis) relative to WT. Each dot represents annotated TU for which antisense relative reads are compared between strains and had a relative value higher than zero. Antisense was separated into two categories: overlapping novel antisense transcripts (n=196 out of 277, blue) and other (n=1466, red). Due lower coverage of the second batch of experiments not all novel antisense transcripts appear as upregulated in *ssu72*Δ. Black diagonal line depicts no change between single and double mutants.
(D) MA plot illustrates transcriptional changes in strain lacking Hos2 compared to WT. Each dot represents annotated transcription unit as described in (Eser et al., 2016), grey reflects no change, red and blue depicts transcripts that were upregulated or downregulated (>1.5 fold, FDR<0.05), respectively. Among upregulated transcripts (94 total) 66 mRNAs were found.
(E) ChIP-qPCR analysis of H4 levels across the *cyr1* and *pom152* gene in WT, *ssu72*Δ, *hos2*Δ and *ssu72*Δ*hos2*Δ. The schematic diagrams show the organisation of *cyr1* or *pom152* loci and positions of primer pairs used for qPCR depicted as back bars. Red arrows indicate the TSS position. The quantification of ChIP-qPCR shows the ratio of IP over the input signal. The error bars represent the standard error of mean (SEM). Results are an average of 3 repeats for WT, *ssu72*Δ and 2 repeats *hos2*Δ, *ssu72*Δ*hos2*Δ, except *pom152* loci where it was repeated twice for all strains.

Figure S4

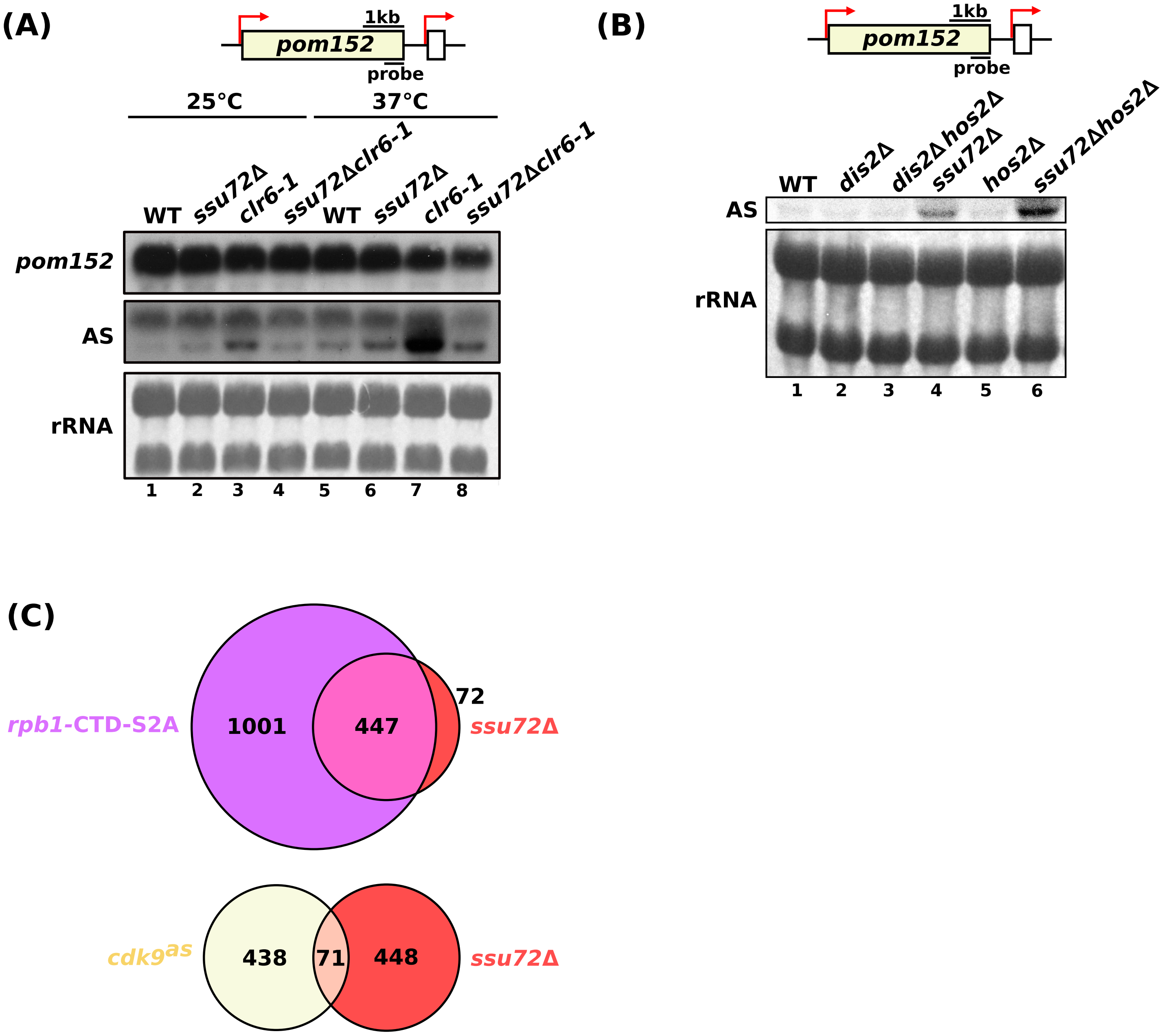

Figure S4. Ssu72 and Rpb1-CTD-S2A mutants accumulate antisense transcripts. Related to Figure 7.

(A) Northern blot analysis for sense and antisense (AS) *pom152* transcripts in indicated strains. To assess RNA levels in temperature-sensitive *clr6-1* strain, RNA was extracted from cells grown at permissive (25°C) and non-permissive (37°C) temperatures. Methylene blue staining was used to visualize rRNA as a loading control.

(B) Northern blot analysis for antisense (AS) *pom152* transcript in indicated strains. Synergistic effect of Hos2 is only associated with the loss of Ssu72 phosphatase. Methylene blue staining was used to visualize rRNA as a loading control.

(C) Venn diagram illustrating the overlap between antisense transcripts dependent on Ssu72, Ser2 phosphorylation of Rpb1 CTD or Cdk9.
